# Identification of regulatory factors promoting embryogenic callus formation in barley through transcriptome analysis

**DOI:** 10.1186/s12870-021-02922-w

**Published:** 2021-03-19

**Authors:** Jingqi Suo, Chenlu Zhou, Zhanghui Zeng, Xipu Li, Hongwu Bian, Junhui Wang, Muyuan Zhu, Ning Han

**Affiliations:** Key Laboratory for Cell and Gene Engineering of Zhejiang Province, Institute of Genetics and Regenerative Biology, College of Life Sciences, Zhejiang University, Zhejiang, 310058 Hangzhou China

**Keywords:** Barley (*Hordeum vulgare*), Callus induction, Auxin response, Plant regeneration

## Abstract

**Background:**

Barley is known to be recalcitrant to tissue culture, which hinders genetic transformation and its biotechnological application. To date, the ideal explant for transformation remains limited to immature embryos; the mechanism underlying embryonic callus formation is elusive.

**Results:**

This study aimed to uncover the different transcription regulation pathways between calli formed from immature (IME) and mature (ME) embryos through transcriptome sequencing. We showed that incubation of embryos in an auxin-rich medium caused dramatic changes in gene expression profiles within 48 h. Overall, 9330 and 11,318 differentially expressed genes (DEGs) were found in the IME and ME systems, respectively. 3880 DEGs were found to be specific to IME_0h/IME_48h, and protein phosphorylation, regulation of transcription, and oxidative-reduction processes were the most common gene ontology categories of this group. Twenty-three *IAA*, fourteen *ARF*, eight *SAUR*, three *YUC*, and four *PIN* genes were found to be differentially expressed during callus formation. The effect of callus-inducing medium (CIM) on *IAA* genes was broader in the IME system than in the ME system, indicating that auxin response participates in regulating cell reprogramming during callus formation. *BBM*, *LEC1*, and *PLT2* exhibited a significant increase in expression levels in the IME system but were not activated in the ME system. *WUS* showed a more substantial growth trend in the IME system than in the ME system, suggesting that these embryonic, shoot, and root meristem genes play crucial roles in determining the acquisition of competency. Moreover, epigenetic regulators, including *SUVH3A*, *SUVH2A*, and *HDA19B/703*, exhibited differential expression patterns between the two induction systems, indicating that epigenetic reprogramming might contribute to gene expression activation/suppression in this process. Furthermore, we examined the effect of ectopic expression of *HvBBM* and *HvWUS* on *Agrobacterium*-mediated barley transformation. The transformation efficiency in the group expressing the *PLTPpro:HvBBM* + *Axig1pro:HvWUS* construct was increased by three times that in the control (empty vector) because of enhanced plant regeneration capacity.

**Conclusions:**

We identified some regulatory factors that might contribute to the differential responses of the two explants to callus induction and provide a promising strategy to improve transformation efficiency in barley.

**Supplementary Information:**

The online version contains supplementary material available at 10.1186/s12870-021-02922-w.

## Background

Genetic transformation has become an essential tool for functional genome research and is a useful technique for crop breeding. A routinely used protocol for the transformation of monocot species depends on in vitro tissue culture. However, many crop cultivars are recalcitrant to regeneration, which is a major bottleneck in plant transformation. Thus, elucidation of the molecular basis of plant regeneration is of great importance for the improvement of plant biotechnology.

A typical *Agrobacterium*-mediated transformation often starts with the induction of pluripotent cells (termed “callus”) from explants cultivated on an auxin-rich callus-inducing medium (CIM). Recent studies have demonstrated that CIM-induced callus formation proceeds via a root meristem-associated pathway [1], displaying an organised spatial expression of root meristem regulator genes such as WUSCHEL-RELATED HOMEOBOX5 (*WOX5*) and SHORT ROOT (*SHR*) [1, 2]. As lateral root development, auxin leads to the degradation of INDOLEACETIC ACID 14 (IAA14) and subsequent activation of AUXIN RESPONSE FACTOR7 (ARF7) and ARF19 [3]; AFR7 and ARF19 then directly enhance the expression of LATERAL ORGAN BOUNDARIES DOMAIN (LBD) proteins, such as LBD16, LBD17, LBD18, and LBD29 [4, 5]. LBD proteins, in turn, activate the expression of a suite of genes that promote cell proliferation and modify cell wall properties [6–8]. Furthermore, auxin promotes cellular pluripotency acquisition via two different pathways, one mediated by WOX11 and LBD16 and the other involving CUP-SHAPED COTYLEDON2 (CUC2) and PLETHORA proteins (PLTs) [9, 10]. Because most researchers have used only *Arabidopsis* for this procedure, it remains unclear whether different species adopt a common mechanism for callus initiation. Our previous work demonstrated that callus induction from root explants employs different strategies in rice and *Arabidopsis* [11]. However, it is still unknown whether the same pathway is involved when an embryo is used as an explant.

Barley (*Hordeum vulgare* L.) is the fourth most abundant cereal crop globally and is widely grown as animal feed and for making malt and brewing wine. The first report of successful *Agrobacterium*-mediated transformation in barley used immature embryos as explants [12]. Although alternative target tissues have been examined for use in barley transformation systems, immature embryos remain the best choice for achieving high transformation efficiencies [13–16]. Moreover, barley transformation is highly genotype dependent. The most responsive genotype is the spring cultivar Golden Promise, and only few barley varieties have been successfully transformed to date [17]. The genes underlying transformability in Golden Promise have been investigated through genetic mapping [18]. Three transformation-amenability loci in Golden Promise (*TFA1*, *TFA2*, *TFA3*) and one locus in mutant 1460 (*TRA1*) were found to be responsible for *Agrobacterium*-mediated transformation in barley [19, 20]. However, the key factors determining explant choice and transformation efficiency remain elusive.

The aim of this study was to provide new insights into the different transcription regulation pathways between calli formed from immature (IME) and mature (ME) embryos through transcriptome sequencing. We outlined a framework of early molecular events behind auxin-induced callus formation in barley, suggesting strategies to enrich the selection range of explants and improve transformation efficiency in barley.

## Results

### Morphologies of calli formed from mature and immature barley embryos

**Fig. 1:**
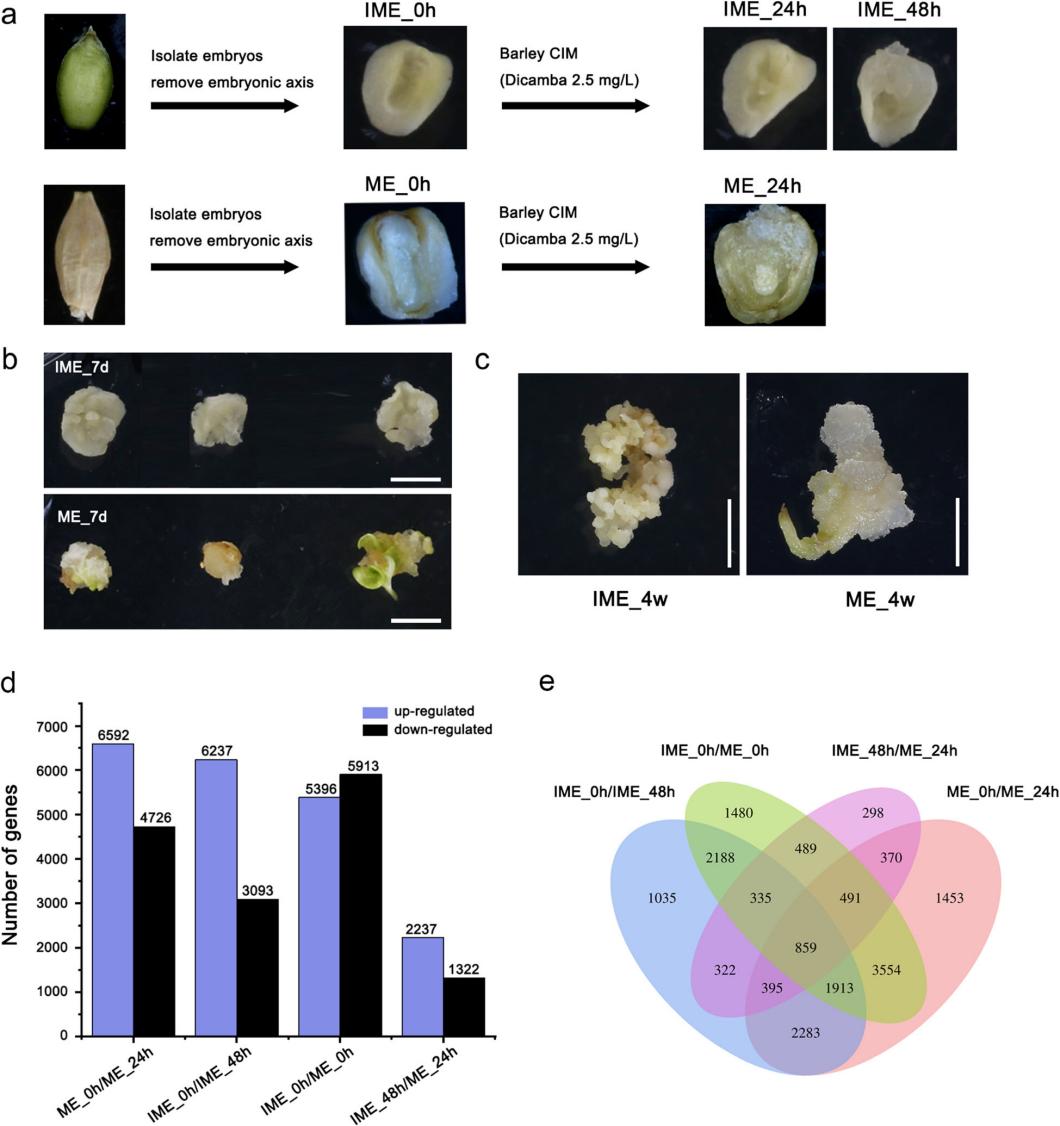
Characteristics of calli formed from immature (IME) and mature (ME) embryos. **a:** Schematic diagram of sample collection for RNA-seq analysis. After surface-sterilisation of mature seeds and embryonic axis removal from immature seeds (14 days post-anthesis), embryos were isolated and cultured on callus-inducing medium (CIM). The samples for RNA-seq were collected at three time points from immature embryo-derived callus (IME_0h, IME_24h and IME_48h), and two time points from mature embryo-derived callus (ME_0h and ME_24h). **b:** Scutellum-induced callus formation in Golden Promise barley after 7 days of culture. Bar =500 μm. **c:** Scutellum-induced callus formation in Golden Promise barley after 4 weeks of culture. Bar =500 mm. **d:** The number of DEGs up- or downregulated during embryo-derived callus formation and between two callus induction systems. **e:** Venn diagram showing overlap and specific DEGs between two samples

Because callus induction and transformation efficiency in barley are genotype-dependent, the model barley variety Golden Promise, which has high callus formation capacity, was selected in this study to investigate the mechanism of callus formation. Immature embryos, approximately 14 days post-anthesis (DPA), and mature embryos, with the embryonic axis removed, were used as explants for callus induction on the same CIM containing 2.5 mg/L dicamba (a synthetic auxin). After 24 h of incubation in the CIM, smooth and watery calli with some degree of normal regeneration (visible shoots) could be seen on the mature seed scutellum. In the IME-induction system, yellow friable calli had emerged from the scutellum peripheral region after 48 h in the CIM (Fig. 1a). Almost all immature embryos had generated calli and maintained a faster proliferation rate after seven days of culture (Fig. 1b). After four weeks of culture, almost all immature embryos, but only a few mature ones, had developed calli; the few calli formed from mature embryos were watery as compared to the dense and granular calli formed from immature embryos (Fig. 1c).

### Global analysis of DEGs expressed in calli derived from mature and immature embryos

To obtain an overview of the mRNA expression profile during callus formation on CIM, we constructed cDNA libraries using five samples, each with three biological replicates. Three of the samples were isolated from IME-derived calli at various time points: IME_0h, IME_24h, and IME_48h. The other two samples were taken from ME-derived calli at different time points: ME_0h and ME_24h (Fig. 1a). Absolute quantitative transcriptome sequencing was then performed using mainstream Unique Molecular Identifier (UMI) labelling technology; through UMI labelling of each sequence, the interference of PCR amplification preference on quantification was eliminated so that the expression abundance of transcripts in the sample could be truly reflected. Raw data totalling 114 Gb were obtained, which contained 759 million paired-end reads. After removing adaptor sequences and low-quality reads, approximately 740 million clean reads remained. Over 99.92% and 97.40% of the clean reads had quality scores of Q20 and Q30, respectively (Additional file 1: Table S1). More than 90.82% of the paired-end reads were mapped to the barley reference genome, with an average of 69.15% for unigenes (Table 1).

**Table 1:** Statistics of the total reads mapped to the reference genome from five libraries

Sample		Valid reads	Mapped reads	Unique mapped reads	Multi-mapped reads	PE mapped reads	Reads map to sense strand	Reads map to antisense strand
IME_0h	1	50,508,092	46,525,933 (92.12%)	34,881,919 (69.06%)	11,644,014 (23.05%)	43,551,514 (86.23%)	19,365,009 (38.34%)	19,429,297 (38.47%)
	2	50,725,082	46,718,943 (92.10%)	35,343,451 (69.68%)	11,375,492 (22.43%)	43,728,232 (86.21%)	19,462,644 (38.37%)	19,535,977 (38.51%)
	3	51,346,982	47,181,924 (91.89%)	35,467,427 (69.07%)	11,714,497 (22.81%)	43,783,770 (85.27%)	19,463,881 (37.91%)	19,543,884 (38.06%)
IME_24h	1	42,482,012	39,600,956 (93.22%)	29,826,685 (70.21%)	9,774,271 (23.01%)	37,024,224 (87.15%)	16,758,583 (39.45%)	16,767,271 (39.47%)
	2	46,361,390	43,046,755 (92.85%)	32,242,450 (69.55%)	10,804,305 (23.30%)	40,242,720 (86.80%)	17,995,697 (38.82%)	18,002,730 (38.83%)
	3	49,289,192	45,811,254 (92.94%)	34,207,322 (69.40%)	11,603,932 (23.54%)	42,805,810 (86.85%)	19,165,613 (38.88%)	19,175,961 (38.91%)
IME_48h	1	50,064,794	45,466,924 (90.82%)	33,987,373 (67.89%)	11,479,551 (22.93%)	42,464,724 (84.82%)	18,904,502 (37.76%)	18,898,466 (37.75%)
	2	52,617,624	48,950,150 (93.03%)	36,748,315 (69.84%)	12,201,835 (23.19%)	45,431,552 (86.34%)	20,474,061 (38.91%)	20,481,498 (38.93%)
	3	51,022,778	47,542,104 (93.18%)	35,553,488 (69.68%)	11,988,616 (23.50%)	44,136,992 (86.50%)	19,950,036 (39.10%)	19,953,944 (39.11%)
ME_0h	1	44,726,072	41,111,321 (91.92%)	30,307,401 (67.76%)	10,803,920 (24.16%)	38,293,348 (85.62%)	17,002,182 (38.01%)	17,013,183 (38.04%)
	2	48,998,490	44,815,937 (91.46%)	33,012,162 (67.37%)	11,803,775 (24.09%)	41,883,774 (85.48%)	18,641,797 (38.05%)	18,639,916 (38.04%)
	3	51,294,828	46,955,145 (91.54%)	34,689,826 (67.63%)	12,265,319 (23.91%)	43,865,372 (85.52%)	19,319,169 (37.66%)	19,322,335 (37.67%)
ME_24h	1	49,828,700	46,579,576 (93.48%)	34,747,312 (69.73%)	11,832,264 (23.75%)	43,617,610 (87.54%)	19,371,793 (38.88%)	19,360,153 (38.85%)
	2	51,117,256	47,940,378 (93.79%)	35,939,931 (70.31%)	12,000,447 (23.48%)	44,326,868 (86.72%)	20,082,418 (39.29%)	20,078,786 (39.28%)
	3	49,956,734	46,882,189 (93.85%)	34,969,303 (70.00%)	11,912,886 (23.85%)	43,922,646 (87.92%)	19,662,673 (39.36%)	19,650,293 (39.33%)

Sample: sequencing library name; Valid reads: the number of reads after UID deduplication; Mapped reads: the number of reads that can be compared to the genome; Unique mapped reads: the number of reads that can only be uniquely aligned to a position in the genome; Multi-mapped reads: the number of reads that can be compared to multiple positions in the genome; PE mapped reads: pair-end sequencing reads are paired to the genome reads; Reads map to sense strand: after UMI deduplication, read comparison to the statistics of the sense strand of the genome; Reads map to antisense strand: after UMI deduplication, the statistics of read alignment to the negative sense strand of the genome

To compare the gene expression profiles correlated with different stages, read numbers were first normalised to the FPKM value. They were then subjected to the usual correlation coefficient (R^2^) and hierarchical clustering analysis. The three biological replicates of all samples showed consistent determinations of transcript abundance with a coefficient (R^2^) greater than 0.87, indicating good repeatability of the sequencing data (Additional file 1: Fig. S1). Further analyses showed that 125,095 transcripts (74.98%) were between 1000 and 5000 bp in length, and 8021 genes (55.21%) were between 1000 and 5000 bp in length (Additional file 1: Table S2).

This criterion (|log_2_fold change| ≥ 1 and *p* value ≤0.05) was used to screen differentially expressed genes (DEGs) (Additional file 2). Since immature embryos start to form calli after 48 h on CIM, while mature embryos start to form calli after 24 h, we chose IME_0h, IME_48h, ME_0h, and ME_24h for further study. The DEGs identified in this study were divided into four groups by pairwise comparisons. The group with the largest number of DEGs was the ME_0h/IME_0h group, with 5396 upregulated genes and 5913 downregulated genes. For both explants, significant gene expression changes were observed during callus formation (Fig. 1d). A Venn diagram showed that 859 DEGs were detected in all four comparison groups (IME_0h/48 h, ME_0h/24 h, IME_0h/ME_0h, IME_48h/ME_24h). In addition to 5450 DEGs that overlapped in two of the comparison groups (IME_0h/48 h, ME_0h/24 h), 3880 and 5868 DEGs were identified specifically in IME- and ME-based induction systems, respectively. A total of 1480 DEGs were only detected in the initial phase of mature and immature embryos before induction (IME_0h/ME_0h) (Fig. 1e).

**Fig. 2:**
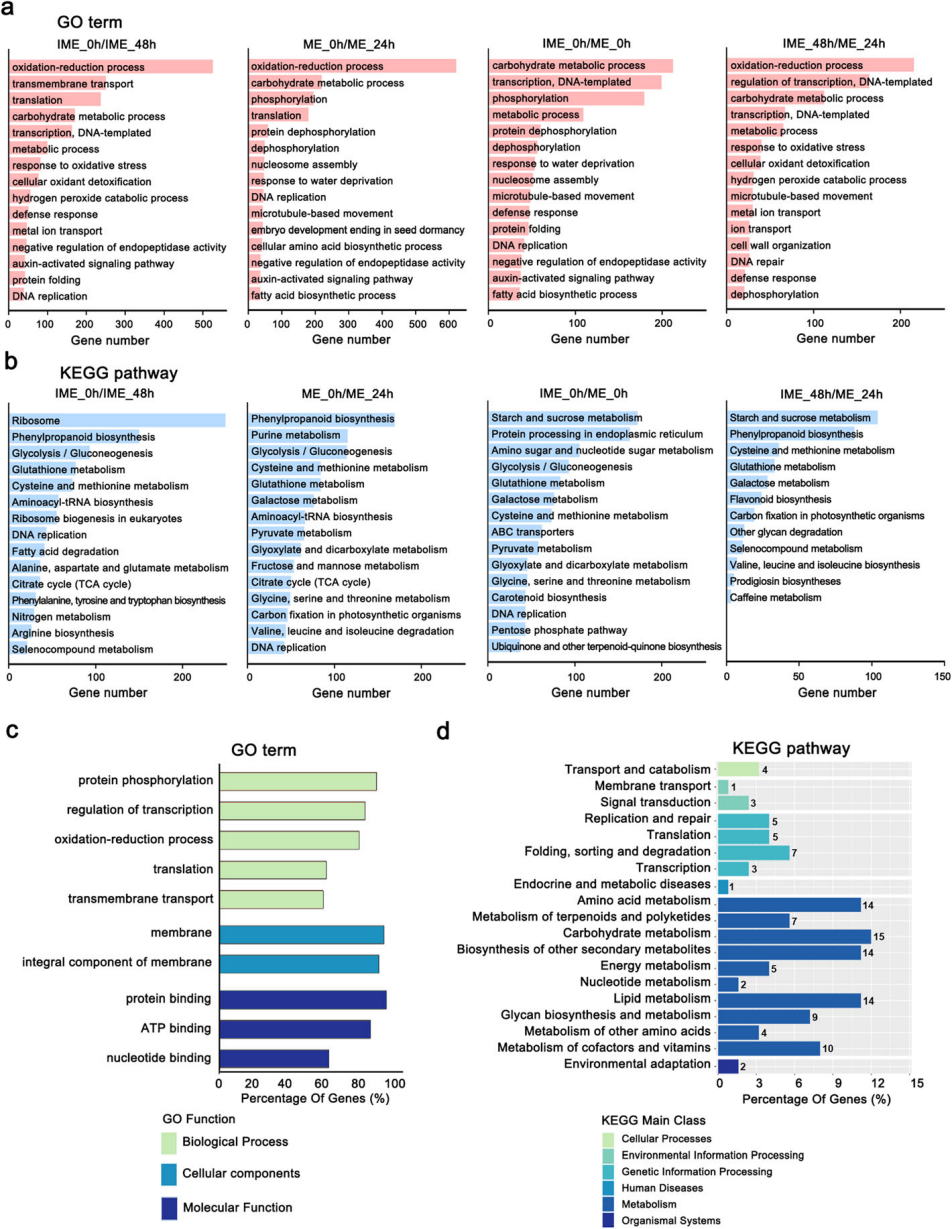
Gene Ontology (GO) and KEGG analysis of differentially expressed genes in different groups. **a:** The selected 15 most enriched GO biological processes categories among DEGs in IME_0h/IME_48h, ME_0h/IME_24h, IME_0h/ME_0h, and IME_48h/ME_24h. **b:** The top 15 most enriched KEGG pathways [67]. *P* value≤0.05, sorted by DEGs number. **c:** The top 10 most enriched GO in the IME-specific DEGs (refer to DEGs only found in the contrast IME_0h/IME_48h, but not included in ME_0h/ME_24h). **d:** The top 19 most enriched KEGG pathway categories in the IME-specific DEGs

Gene ontology (GO) analysis showed that oxidative-reduction process was the most common GO category of DEGs in IME_0h/IME_48 h, ME_0h/ME_24 h, and IME_48h/ ME_24h, while DEGs in IME_0h/ ME_0h were mainly involved in carbohydrate metabolic process, transcription, and phosphorylation etc. (Fig. 2a). KEGG analysis revealed that DEGs related to phenylpropanoid biosynthesis and glycolysis/gluconeogenesis were enriched in IME_0h/IME_48 h, ME_0h/ME_24 h, and DEGs for starch and sucrose metabolism were represented in IME_0h/ ME_0h and IME_48h/ ME_24h (Fig. 2b).

In addition, we separately analysed IME-specific DEGs, which refer to DEGs only found in the contrast IME_0h/IME_48h, but not included in ME_0h/ME_24h. GO analysis showed that protein phosphorylation, regulation of transcription, oxidative-reduction process, membrane, and protein, ATP, and nucleotide binding were the most common GO categories of DEGs specific to the IME system (Fig. 2c). Amino acid metabolism, carbohydrate metabolism, lipid metabolism, and biosynthesis of other secondary metabolites were the most enriched pathways associated with IME-specific DEGs (Fig. 2d).

### Differential expression of transcriptional regulators involved in callus induction

Transcription factors (TFs) play critical roles in embryogenic callus formation by regulating cell proliferation and cell fate reprogramming [21, 22]. The Plant Transcription Factor Database PlantTFDB (http://planttfdb.cbi.pku.edu.cn) was used to sequence blast and annotate the TFs in barley associated with callus initiation in our dataset.

Dramatic changes in the expression of TFs occurred in both callus induction systems. Four hundred and thirty TFs were identified in the IME_0h/IME_48h group, and 472 TFs were identified in the ME_0h/ME_24h group (Additional file 2). Among them, B3, bHLH, NAC, bZIP, and MYB-related TFs ranked in the top five IME_0h/IME_48h group (Additional file 1: Fig. S2a, Additional file 2). In addition, bHLH, NAC, ERF, bZIP, and MYB family members were among the differentially expressed TFs enriched in the ME_0h/ME_24h group (Additional file 1: Fig. S2b, Additional file 2). In the IME_0h/IME_48h group, the transcript levels of *HD-ZIP1* (HORVU4Hr1G078410), *PRE5* (HORVU4Hr1G075340), *LBD16* (HORVU0Hr1G017670), WUSHEL (*WUS*, HORVU3Hr1G085050), and *ESE3* (HORVU7Hr1G029870) increased, whereas the levels of *ERF48* (HORVU1Hr1G063100), *SRS-like* (HORVU6Hr1G084070), and *C2H2-like* (HORVU5Hr1G112900) decreased during callus induction (Additional file 1: Fig. S2c). In the ME_0h/ME_24h group, the transcript levels of *LBD29* (HORVU4Hr1G080160), *bHLH-like* (HORVU3Hr1G030760), and *NAC071* (HORVU1Hr1G049840) increased, whereas the levels of *SRS-like* (HORVU6Hr1G084070), *bZIP-like* (HORVU4Hr1G021720), and *HSF-like* (HORVU2Hr1G040680) appeared to decrease during callus induction (Additional file 1: Fig. S2d).

**Fig. 3:**
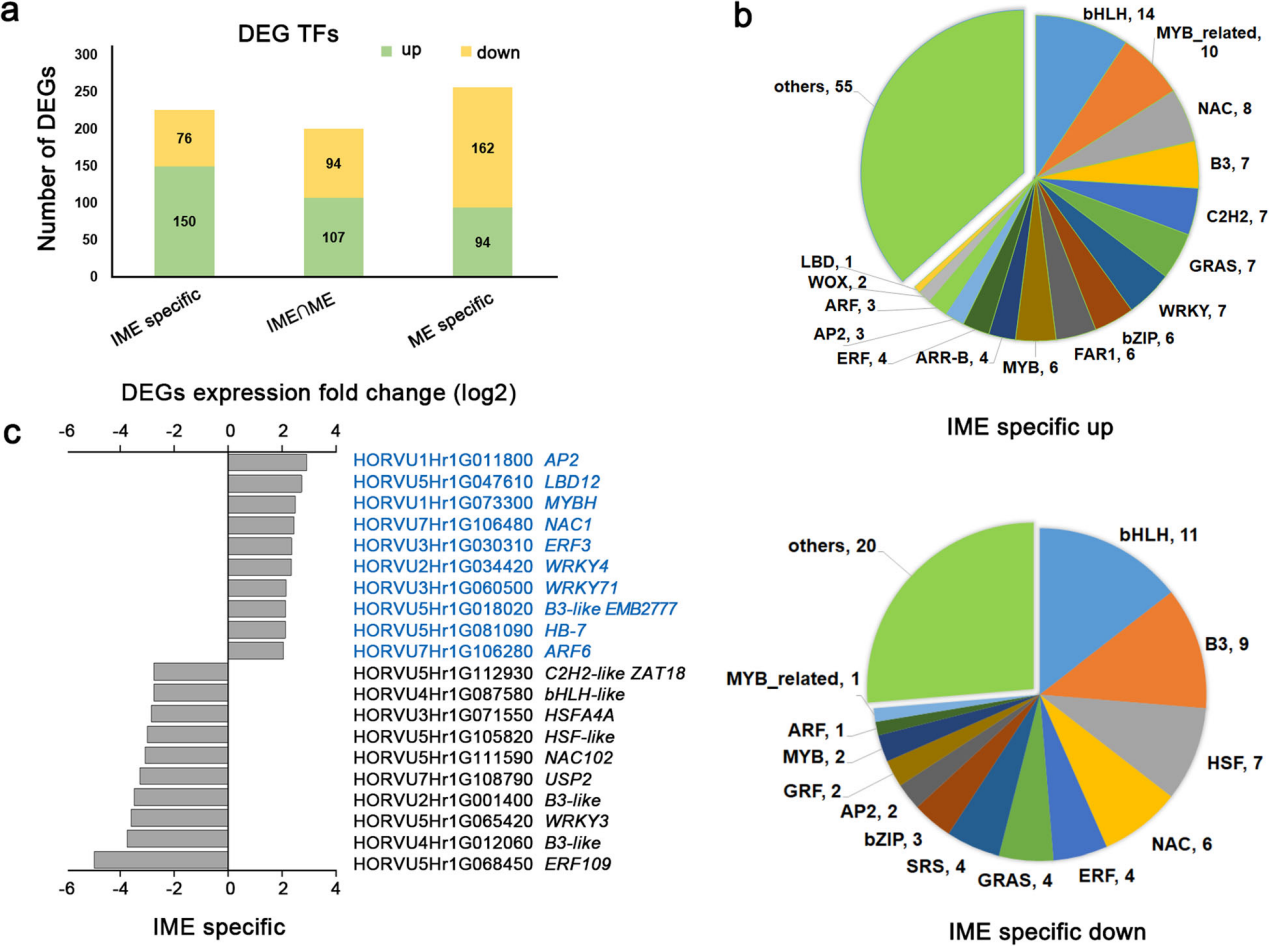
Expression of a set of callus-inducing medium (CIM)-induced transcription factors (TFs) during - callus formation from immature and mature embryos. **a:** The number of differentially expressed TFs detected only in the IME system (IME specific), only in the ME system (ME specific), and in both systems (IME∩ME). **b:** Upregulated (upper) and downregulated (lower) TF families specific to the IME system. Numbers represent the gene members associated with a given TF family. **c:** The top 10 differentially expressed TFs in the IME system (IME specific). Genes marked in blue are upregulated TFs, and TFs marked in black are downregulated

Since we were interested in identifying TFs determining embryonic callus formation, we further analysed TF transcripts that were differentially regulated only in the IME group. A set of 226 TF genes, including 150 upregulated and 76 downregulated genes, were identified as DEGs, specifically during IME-derived callus induction (IME-specific). These TFs may contribute to the differential response of the two explants to callus induction (Fig. 3a). In addition, bHLH, NAC, MYB, B3, and HSF family members were among the differentially expressed transcriptional factors enriched in this group (Fig. 3b). Among them, significantly differentially expressed TFs, with a fold change greater than 4.5 (*p* value < 0.05), found explicitly in the IME system, are shown in Fig. 3c. The transcript levels of *AP2* (HORVU1Hr1G011800), *LBD12* (HORVU5Hr1G047610), *MYBH* (HORVU1Hr1G073300), *NAC1* (HORVU7Hr1G106480), and *ERF3* (HORVU3Hr1G030310) increased, whereas the levels of *ERF109* (HORVU5Hr1G068450), *B3-like* (HORVU4Hr1G012060), and *WRKY3* (HORVU5Hr1G065420) decreased during callus induction.

### Auxin signalling during CIM-mediated callus formation

**Fig. 4:**
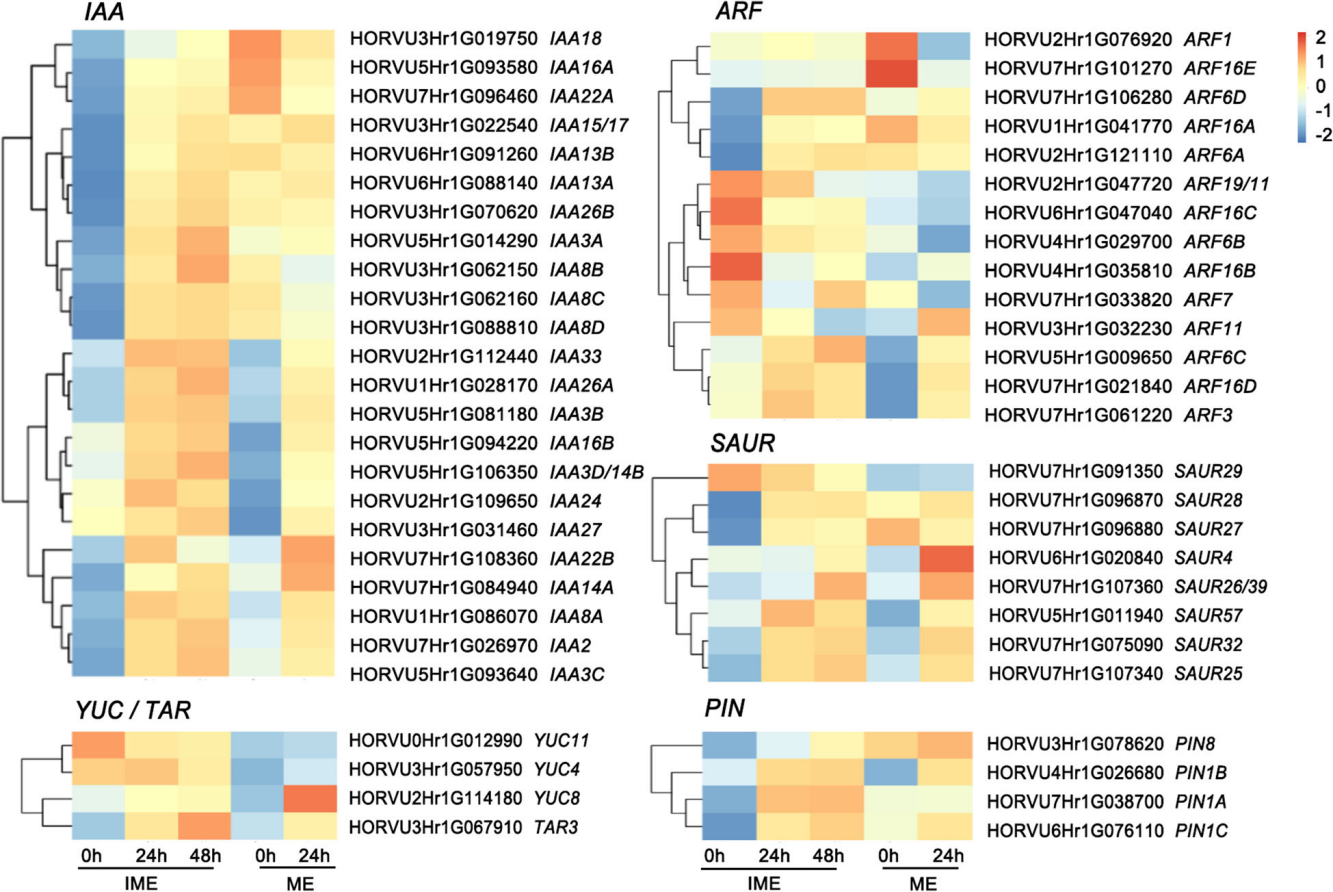
Expression of genes involved in the auxin pathway during callus-inducing medium (CIM)-mediated callus formation. The expression levels were visualised by using OmicStudio tools at https://www.omicstudio.cn/tool based on RNA-seq datasets (Additional file 4). Numbers beneath the heat map indicate the relative expression intensities, and the higher expression intensities are indicated by more reddish colours. Genes are grouped by auxin response, biosynthesis, and transport genes. Note that only genes with FPKM > 1 are shown

Auxin has been reported to play vital roles in promoting cell proliferation and reprogramming during callus formation in tissue culture [23–25]. We examined the transcriptional profiles of genes related to auxin response, biosynthesis, and transport. Twenty-three *IAA*, fourteen *ARF*, eight *SAUR*, three YUCCA (*YUC*), and four PIN-FORMED (*PIN*) genes were found to be differentially expressed during callus formation (Fig. 4). The effect of CIM on *IAA* genes was broader in the IME system than in the ME system. Twenty-two *IAA* candidate genes exhibited a significant increase in expression level when IME was used as the explant; ten *IAA* genes were found to be upregulated (more than 3 times) in the ME group. Most of the analysed *ARF* genes showed differential expression patterns between the two groups (Fig. 4). Notably, candidate genes *ARF11* (HORVU3Hr1G032230) and *ARF16B* (HORVU4Hr1G035810) exhibited different patterns between the two groups, and two putative *ARF6* genes (HORVU2Hr1G121110 and HORVU7Hr1G106280) were upregulated in the IME group but remained unchanged in the ME group. These data indicate that genes associated with auxin response cooperate in regulating cell reprogramming during auxin-induced callus formation. The expression of candidate genes, including *ARF11*, and *ARF16B* was detected using quantitative reverse transcription PCR (qRT-PCR). The results are shown in Additional file 1: Fig. S3.

### Changes in the expression of key developmental genes for embryo, root, and shoot meristems during callus formation

**Fig. 5:**
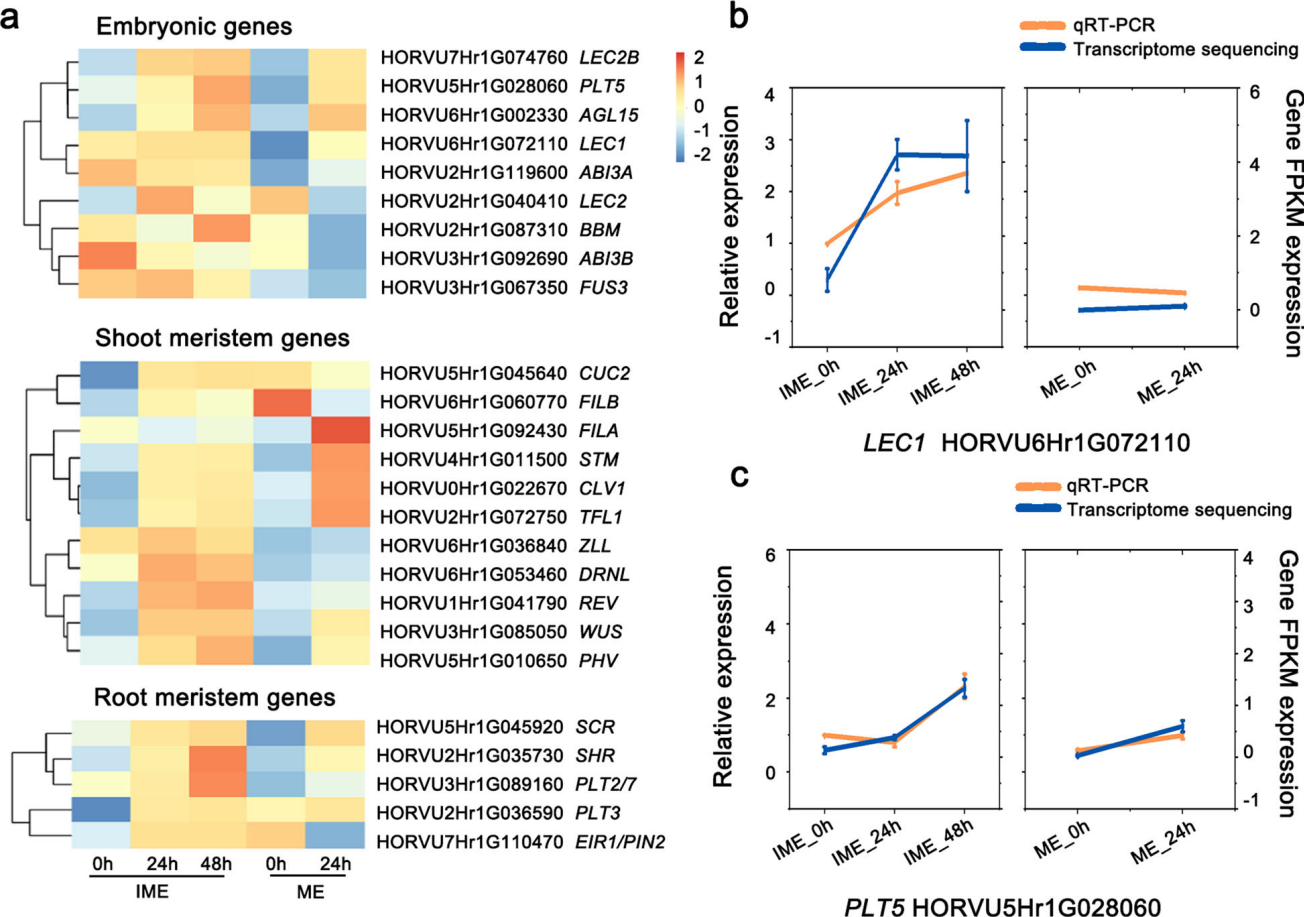
Heat map showing expression changes of key developmental genes for embryos and meristems during callus induction. The expression levels were visualised by using OmicStudio tools at https://www.omicstudio.cn/tool based on RNA-seq datasets (Additional file 4). **a:** Clustering display of expression intensities of the embryonic, shoot, and root meristem genes based on RNA-seq datasets. **b:** The transcript levels of *LEC1* and *PLT5* in five samples were revealed by qRT-PCR and RNA-seq data. The data shown are means ± S.D. of three biological replicates

Cells are thought to dedifferentiate and acquire competency when they divide to form calli [2, 26]. To assess the embryogenic character of the embryo-derived calli, marker genes presenting embryo, root, and shoot meristems were analysed. Among the nine putative embryonic genes, the transcripts of FUSCA3 (*FUS3,* HORVU3Hr1G067350) and ABSCISIC ACID-INSENSITIVE 3A (*ABI3A*, HORVU2Hr1G119600) were higher in the IME system than in the ME system. Notably, BABY BOOM (*BBM*, HORVU3Hr1G089160) and *LEC1* (HORVU6Hr1G072110) displayed significant increases in expression levels during IME-derived callus formation but were not activated in the ME system (Fig. 5a). The results of qRT-PCR verification were consistent with those of RNA-Seq (Fig. 5b, Fig. 5c).

Among the 11 upregulated shoot apical meristem (SAM) genes, two genes were only upregulated in the IME system. In addition, transcription of *CUC2* (HORVU5Hr1G045640) and *FILB* (HORVU6Hr1G060770) was rapidly activated during callus formation in the IME system but was suppressed in the ME system. Remarkably, WUSCHEL (*WUS*, HORVU3Hr1G085050) exhibited more significant growth trends in the IME system than in the ME system. Five root apical meristem (RAM) genes were upregulated, four of which overlapped in the two groups. *PLT2* (HORVU3Hr1G089160) displayed a significant increase in expression during IME-derived callus formation but was not activated in the ME system. The increases in *SHR* (HORVU2Hr1G035730) and *PLT3* (HORVU2Hr1G036590) transcripts were greater in the IME system than in the ME system; *EIR1/PIN2* (HORVU7Hr1G110470) exhibited the opposite pattern (Fig. 5a).

### Verification of transcriptional regulators might promote embryonic callus formation and transformation

**Fig. 6:**
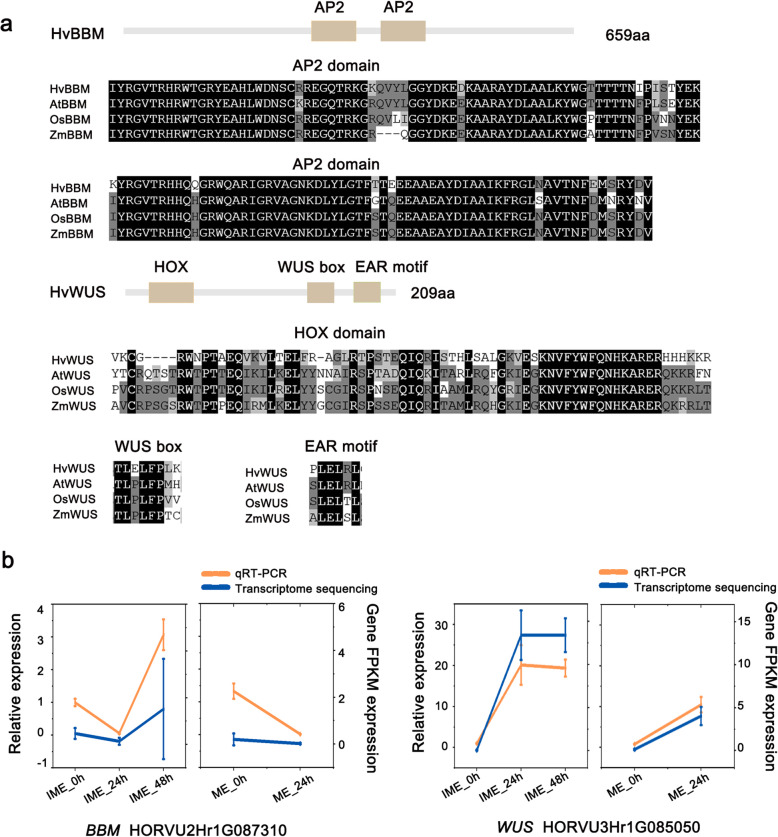
Identification of *BBM* and *WUS* candidate genes in barley and their expression response to callus-inducing medium (CIM). **a:** Sequence alignment and domain analysis of *BBM* and *WUS* in *Arabidopsis*, rice, maize, and barley. **b:** The transcript levels of *BBM* and *WUS* in the five samples were revealed by qRT-PCR and RNA-seq data. The data shown are means ± S.D. of three biological replicates

This study identified one *BBM* gene and one *WUS* gene in barley (Additional file 1: Fig. S4)—HORVU2Hr1G087310 (termed *BBM*) and HORVU3Hr1G085050 (termed *WUS*). These genes exhibited differential expression patterns between the two systems (Fig. 6b); thus, we investigated them further in our study. HvBBM contains two AP2 DNA-binding domains, which are highly consistent with the amino acid sequences of genes in maize, rice, and *Arabidopsis thaliana* (Fig. 6a). The barley WUS, an ortholog of *the Arabidopsis* stem cell regulator WUS [27, 28], contains a HOX domain, a WUS box, and an EAR motif (Fig. 6a). Phylogenetic trees showed that the candidate barley *BBM* was closer to the other two monocot genes, and the candidate barley *WUS* was closer to the WUS of the dicot *Arabidopsis thaliana* (Additional file 1: Fig. S4).

To verify the reliability of the sequencing data, qRT-PCR was performed to detect the gene expression levels during the early stages of callus formation. The expression level of *HvBBM* increased gradually when immature embryos were used as explants but decreased during callus induction when mature embryos were used (Fig. 6b). This suggests that the *BBM* gene contributes to the differential response of explants to CIM. As for *WUS*, a greater increase in transcription was observed in the IME system than in the ME system (Fig. 6b).

We also analysed candidate gene *LEC1*. The protein encoded by LEC1 contained one CCAAT binding factor (CBF), with an amino acid sequence highly conserved among barley and other species, such as maize, rice, and *Arabidopsis thaliana* (Additional file 1: Fig. S5a). The transcript levels of *LEC1* in the five samples were revealed through qRT-PCR, the results of which were consistent with the RNA-seq data (Fig. 5b). Phylogenetic trees showed that the candidate barley LEC1 was closer to the other two homologs in monocots (Additional file 1: Fig. S5b).

### Ectopic expression of *HvBBM* and *HvWUS* improved genetic transformation efficiency

**Fig. 7:**
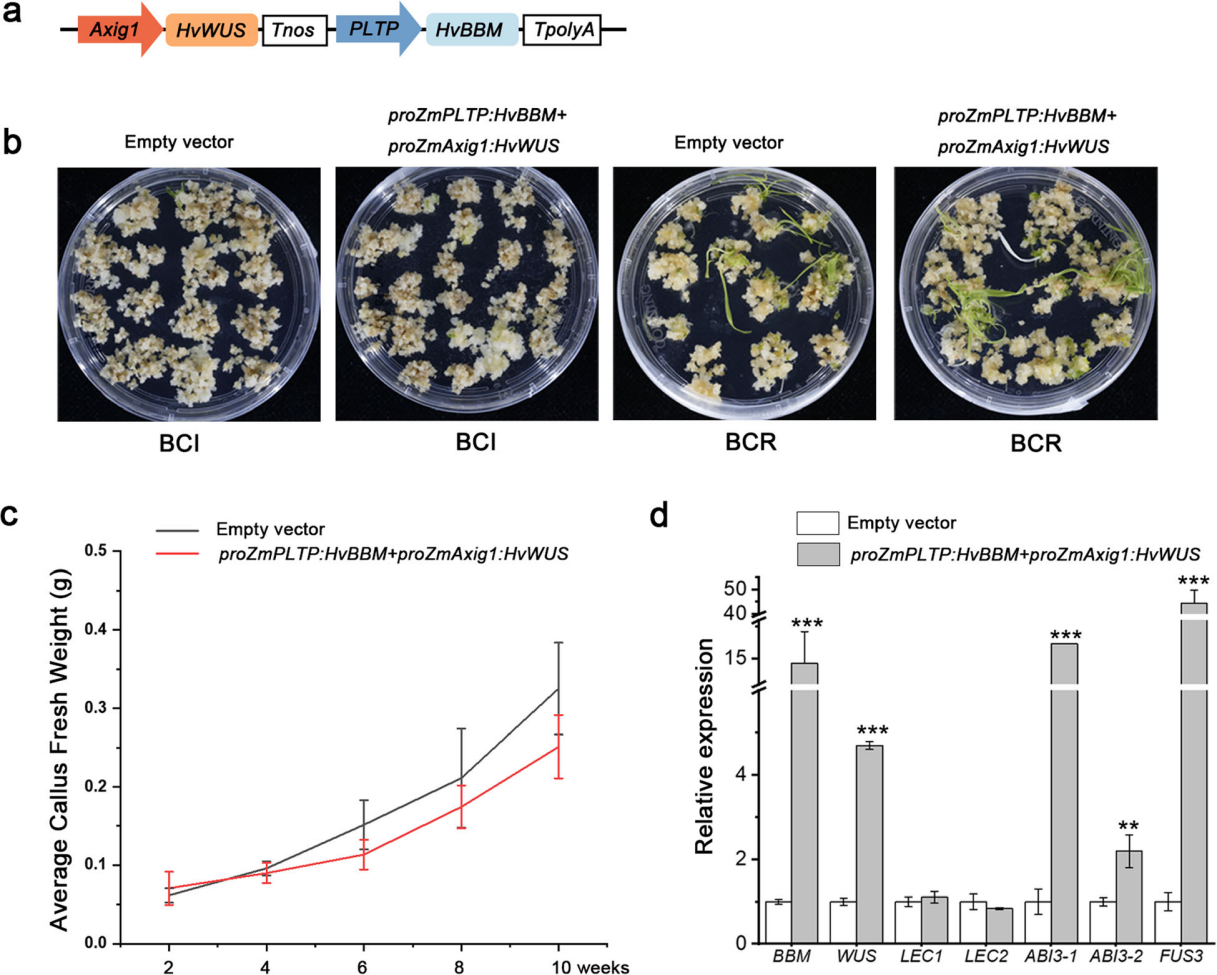
The effect of *BBM* and *WUS* ectopic expression on callus-inducing medium (CIM)-induced callus formation and transformation efficiency. **a** Schematic representation of the construct used for *Agrobacterium*-mediated barley transformation. The *proZmPLTP:HvBBM + proZmAxig1:HvWUS* construct contained two cassettes: the first one included the maize phospholipid transferase promoter (*proZmPLTP)* driving *HvBBM* with a Nos terminator, and the second one included the maize *Axig1* promoter (*proZmAxig1*) driving *HvWUS* with a Nos terminator. **b** The callus-forming and plant regeneration phenotype after *Agrobacterium* inoculation. The group using an empty vector was set as control. **c** Fresh weight analysis of callus in the control and *proZmPLTP:HvBBM + proZmAxig1:HvWUS* transformation group. Error bars indicate the SE of the mean (*n* = 30). The experiments were performed in three independent replicates. **d** The effect of *BBM* and *WUS* ectopic expression on the genes in the *LEC1-ABI3-FUS3-LEC2* network. The data shown are means ± S.D. of three biological replicates.**,* P* < 0.05; ***, *P *<0.01 (Student’s *t*-test).

Overexpression of maize (*Zea mays*) *BBM* and maize *WUS2* genes stimulated transformation in numerous previously non-transformable inbred maize lines, immature sorghum embryos, sugarcane calli, and indica rice callus [29, 30]. In this study, the expression patterns of *BBM* and *WUS* were found to vary between IME- and ME-derived callus induction (Fig. 6b). To further investigate the effect of ectopic expression of *BBM* and *WUS* on callus formation and transformation efficiency in barley, two constructs were designed, each of which contained two expression cassettes: a maize *PLTP* promoter driving a maize *BBM* (*ZmBBM*) or a barley *BBM* (*HvBBM*) combined with a maize *Axig1* promoter driving a maize *WUS* (*ZmWUS*) or a barley *WUS* (*HvWUS*) (Fig. 7a). The generated vector was presented as *proZmAxig1:HvWUS + proZmPLTP:HvBBM*. Using immature embryos as explants, *Agrobacterium*-mediated transformation was carried out. After *Agrobacterium* inoculation, calli were selected on hygromycin-containing callus induction medium and then transferred to shoot-inducing medium (SIM) for plantlet regeneration (Fig. 7b). The callus proliferation rate was measured by the fresh weight of callus, and no significant phenotypic changes in regenerated plantlets were observed after the delivery of the *proZmAxig1:HvWUS + proZmPLTP:HvBBM* construct (Fig. 7c, Additional file 1: Fig. S6).

Transformation of the *proZmAxig1:HvWUS* + *proZmPLTP:HvBBM* construct created transgenic plantlets at a frequency of 24.80%. When *proZmAxig1:ZmWUS + proZmPLTP:ZmBBM* was used, transgenic plantlets were produced with a mean frequency of 4.00%, compared with a frequency of 7.32% for the empty vector. In particular, the regeneration frequency increased from 7.32 to 24.8%, indicating that the effect of *HvBBM* and *HvWUS* on the transformation efficiency might depend on its promotion of plant regeneration (Table 2).

**Table 2:** The effect of *BBM* and *WUS* on *Agrobacterium*-mediated barley transformation using immature embryos as explants

Developmental Gene Expression Cassettes	No. of explants	No. of regenerated plants	Regeneration Freq.	No. of T_**0**_	Transformation Freq.
*pCAMBIA1305* EV	615	48	7.80%	45	7.32%
*proZmPLTP:ZmBBM* +*proZmAxig1:ZmWUS*	575	27	4.7%	23	4.00%
*proZmPLTP:HvBBM* +*proZmAxig1:HvWUS*	613	155	25.29%	152	24.8%

*Agrobacterium* (strain EHA105)-mediated barley transformation was performed using immature embryos as explants. The *proZmPLTP:HvBBM + proZmAxig1:HvWUS* construct contains two cassettes, the first of which includes a maize phospholipid transferase promoter (*proZmPLTP)* driving *HvBBM*, and the second includes the maize *Axig1* promoter (*proZmAxig1*) driving the *HvWUS*. *The pCAMBIA1305* empty vector (EV) was used as a control. Regeneration Freq. was estimated as the No. of regenerated plants divided by the No. of the explants, and the transformation frequency was calculated as No. of T_0_ divided by No. of explants

According to previous research, *BBM* is known to activate the LEC1-ABI3-FUS3-LEC2 network to induce somatic embryogenesis [31]. We then detected the expression of these genes downstream of the *BBM*. The transcript levels of ABSCISIC ACID-INSENSITIVE3 (*ABI3*) and FUSCA3 (*FUS3*) were significantly increased in calli co-expressing *HvBBM* and *HvWUS* (Fig. 7d).

### Transcriptional changes of genes regulating DNA methylation and histone modification

**Fig. 8:**
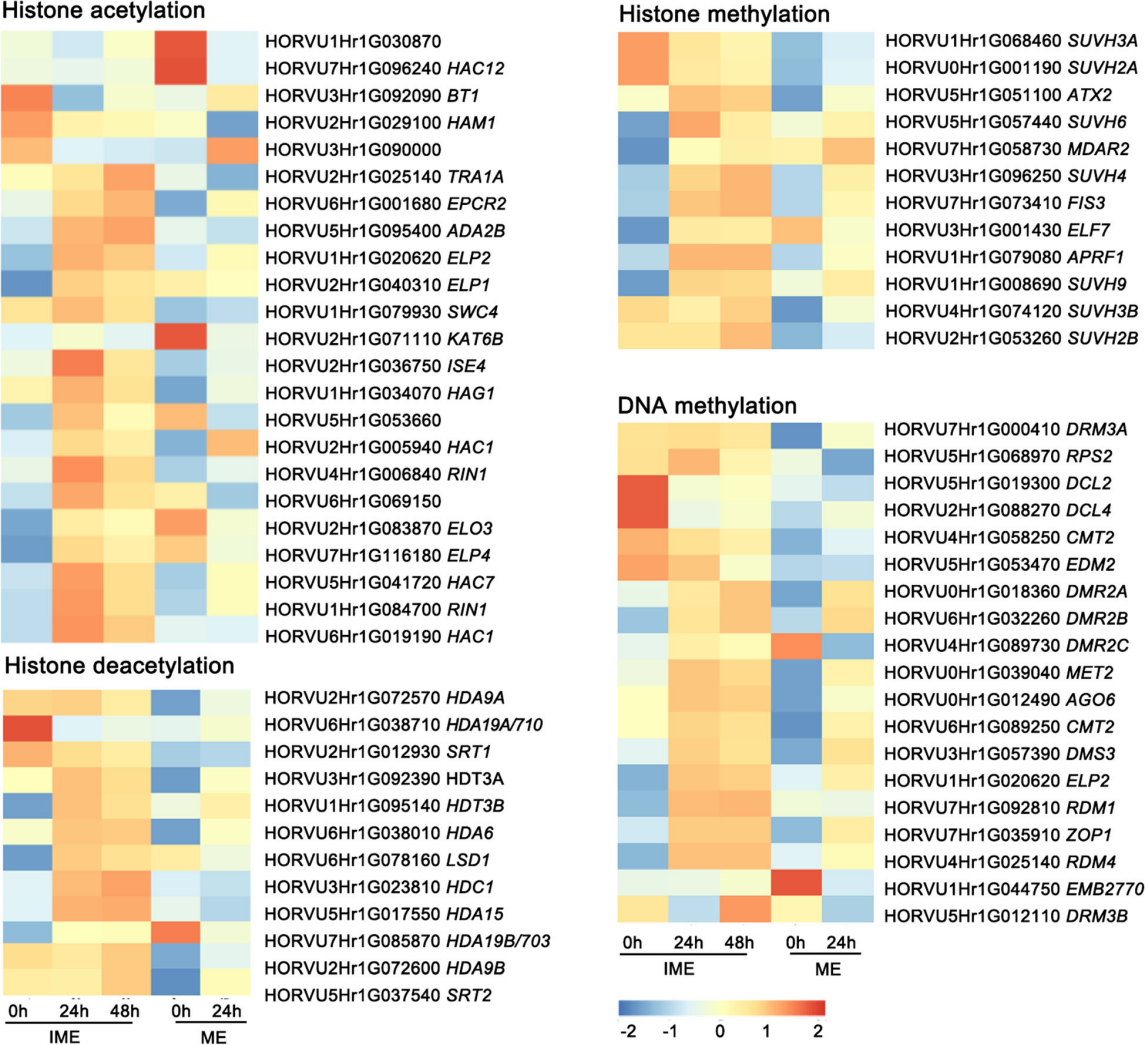
Transcriptional changes of genes regulating DNA methylation and histone modification. The expression levels were visualised by using OmicStudio tools at https://www.omicstudio.cn/tool based on RNA-seq datasets (Additional file 4). Numbers beneath the heat map indicate the relative expression intensities, and the higher expression intensities are indicated by more reddish colours. Note that only genes with FPKM > 1 are shown

Epigenetic reprogramming plays an essential role in callus induction, somatic embryogenesis, and totipotency acquisition [32]. Among the putative histone methyltransferases, *SUVH4* (HORVU3Hr1G096250) was activated in both systems, and HORVU1Hr1G008690 (*SUVH9*) was only induced in the IME system. *SUVH3A* (HORVU1Hr1G068460) and *SUVH2A* (HORVU0Hr1G001190) candidate genes were downregulated in the IME system but remained low level in the ME system (Fig. 8, Additional file 1: Fig. S3).

Two genes associated with histone acetylation were upregulated in the two systems (*RIN1*, *HAC7*) and *ELP2* (HORVU1Hr1G020620) were specifically induced in the IME system. The expression of *HAC12* (HORVU7Hr1G096240) was suppressed in the ME system (Fig. 8). Two genes involved in histone deacetylation, *HDA19/703* (HORVU7Hr1G085870) and *LSD1* (HORVU6Hr1G078160), were upregulated in the IME system but remained suppressed or unchanged in the ME system.

As a critical component of epigenetic regulation, DNA methylation-related genes display significant changes during callus induction [32]. Six upregulated genes were found in both systems (*DRM2A*, *DRM2B*, *MET2*, *AGO6*, *CMT2*, and *DMS3*). *ELP2* (HORVU1Hr1G020620) was only activated in the IME system (Fig. 8). Taken together, these data suggest that epigenetic reprogramming might play an essential role in regulating gene expression during auxin-induced callus formation.

## Discussion

Barley is one of the most recalcitrant crops for tissue culture among the major cereals. Immature embryos are commonly used as explants for barley transformation. However, these embryos need to be dissected out individually from developing seeds, which requires significant labour and is subject to seasonal constraints. In contrast, although mature embryos are easily accessible, it is challenging to form callus with regenerative potential using mature embryos in tissue culture. In this study, we explored global transcriptional changes during embryo-derived callus induction and identified some potential factors that might contribute to the differential responses of the two types of explants to exogenous application of auxin.

### Global transcriptional changes during auxin-induced callus formation in barley

Our RNA-seq data showed that incubation of embryos on auxin-rich medium caused dramatic changes in gene expression profiles within 48 h. A total of 9330 and 11,318 DEGs were found in the IME and ME systems, respectively. Most of the genes overlapped significantly, suggesting that these genes are generally associated with callus formation in different systems. Nearly 11.09% and 12.84% of DEGs were found to be specific to IME_0h/IME_48h and ME_0h/ME_24h, respectively (Fig. 1e). The IME_0h/ME_0h group contained the largest number of DEGs (Fig. 1d, Fig. 2a), indicating that carbohydrate metabolic processes and gene transcription during grain development determine the nature of explants and their responses to the callus induction medium. Genes involved in various activities, such as protein phosphorylation, regulation of transcription, and the oxidation-reduction process, are enriched during IME-based callus formation (Fig. 2c). A previous study revealed that protein tyrosine phosphorylation might play an important regulatory role in phytohormone-stimulated cell proliferation [33, 34]. Furthermore, TOR kinase activated by sugar was found to phosphorylate and stabilise E2Fa proteins, which transcriptionally activate S-phase genes during callus formation [35]. The phosphorylation of E2Fa is also known to enhance its transcriptional activity [36]. These results indicate that protein phosphorylation participates in the regulation of cell proliferation during callus formation. Furthermore, we showed that the oxidation-reduction process was significantly enriched in most comparisons. Redox homeostasis is thought to be essential for sustaining metabolism, growth, and stem cell maintenance and differentiation [37]. Thioredoxin-dependent redox modification has been reported to regulate de novo shoot initiation via ROS homeostasis, which explains the natural variation in plant regeneration [38]. Thus, it will be interesting to further explore the importance of ROS homeostasis in callus formation and regenerative competence.

### Effect of auxin signalling on CIM-induced callus formation in barley

The plant hormone auxin is well established as an efficient inducer of callus formation. This study showed that the ability to form calli and its auxin signalling pathway varies between mature and immature embryos. The effect of CIM on *IAA* genes was more significant in the IME system than in the ME system, indicating that immature embryos are more sensitive to exogenous auxin supplementation. A total of 23 *IAA*, 14 *ARF*, 8 *SAUR*, 3 *YUC*, and 4 *PIN* genes were found to be differentially expressed during callus formation (Fig. 4), suggesting that auxin response and transport processes are necessary for the regulation of cell reprogramming during auxin-induced callus formation.

Studies in *Arabidopsis* have shown that during CIM-mediated callus formation, auxin signalling is transduced via ARF transcription factors, especially ARF7 and ARF19, to activate the expression of LBD family transcription factors [3–5], thereby inducing E2Fa to promote cell cycle reentry [39]. As such, we compared the transcription of *LBD* genes in our sequencing samples, which revealed that different combinations of *LBD16B* and *LBD29A/B* were upregulated in the two systems (Additional file 1: Fig. S7). Therefore, we suppose that LBD proteins act downstream of ARF factors to reinforce callus formation through cell cycle regulators or cell wall modification [6, 7, 39].

### Embryonic genes play crucial roles in determining the acquisition of competency

We focused our analysis on key developmental genes for embryos and root and shoot meristems to determine the molecular identity of different explants and their derived calli. Root meristem regulator genes such as *PLT2* and *SHR* were shown to be significantly upregulated in the IME system, supporting the finding that calli that develop on CIM have histological features resembling the root meristem [1, 10, 40].

Although both types of explants produce calli on auxin-rich medium, only immature embryo-derived embryonic calli acquire high regeneration potential in SIM [41]. Recent studies have shown that the embryonic nature of explants is a prerequisite for somatic cell reprogramming [42]. Ectopic overexpression of embryonic regulators or meristematic regulators induces callus formation in various plant species, illustrating that activation of undifferentiated cell fate is sufficient to drive unorganised cell proliferation [27, 29, 43–46]. Our transcriptome sequencing data showed that embryonic marker genes, such as *BBM* and *LEC1*, were rapidly induced by auxin, specifically in the IME system, and *FUS3* and *ABI3* maintained high-level transcription (Fig. 5a). These genes function as transcriptional activators during embryogenesis. When either of these transcription factors is ectopically expressed in *Arabidopsis*, the resulting plants produce embryonic calli on phytohormone-free medium [31, 43, 44, 46–48]. These results demonstrate that TFs involved in embryonic development are required for cell fate reprogramming, which is necessary for embryonic callus formation in CIM. As for shoot meristem genes, most of them exhibited strong or mild expression in the IME system, and only three of them were induced in the ME system (Fig. 5a). Notably, a *WUS* candidate gene was found to be significantly activated within 24 h of culture on CIM. Furthermore, the transcriptional level of *WUS* increased more in the IME system than in the ME system (Fig. 5a). The induction of *WUS*, the organising centre regulator, has been reported to participate in the most critical events during shoot induction from the callus on SIM, which is controlled by the interaction between auxin and cytokinin [49, 50]. In contrast, auxin-induced *WUS* expression is required for the activation of the embryonic regulators *LEC1* and *LEC2* during somatic embryogenesis [51]. *LEC1* and *LEC2*, combined with *BBM* and *AGL15*, form highly interconnected transcriptional networks and promote the expression of *YUCs*, *TAA1*, and *IAA30* to modulate auxin biosynthesis and signalling [21]. Therefore, we propose that activated *WUS* expression during CIM incubation might confer pluripotency to callus cells through multiple pathways.

### Overexpression of *BBM* and *WUS* enhances transformation efficiency through regulation of regeneration potential

*BBM* is an AP2/ERF transcription factor preferentially expressed during embryogenesis and seed development [45], whereas *WUS* is a homeodomain-containing transcription factor expressed in the stem cell organising centre of shoot meristems as well as in several callus lines [27, 28, 52]. Previous studies have demonstrated that overexpression of *BBM* induces embryonic callus in *Arabidopsis* [45] and several crop and tree species [53], and overexpression of *WUS* generates callus and somatic embryos in *Arabidopsis* [54]. These results indicate that the functions of *BBM* and *WUS* in promoting embryogenesis or embryonic callus formation might be conserved across dicots and can be used to increase the efficiency of callus induction. Maize *BBM* and *WUS2* have been successfully applied to stimulate transformation in maize, sorghum, sugarcane, and *indica* rice [55]. Considering the pleiotropic effects, such as phenotypic abnormalities and sterility, induced by the constitutive expression of maize *BBM* and *WUS2*, callus-expressed promoters (*Zm-PLTPpro*) and auxin-inducible promoters (*Zm-Axig1pro*) have been used to drive the expression of *BBM* and *WUS2*, and transgenic plants have been obtained through somatic embryos [29]. We tested this method by replacing maize genes with barley *BBM* and *WUS* and generated healthy and fertile transgenic plants (Additional file 1: Fig. S6). Co-expression of barley *BBM* and *WUS* significantly increased the efficiency of transformation by approximately three times (Table 2). In the process of *Agrobacterium* cocultivation and subsequent selection, no significant changes were observed in callus-induction capacity, and the callus proliferation ability reflected by fresh weight was even lower upon co-expression of *PLTPpro:HvBBM* and *Axig1pro:HvWUS* (Fig. 7c). However, the plant regeneration capacity was significantly increased in the callus expressing *PLTPpro:HvBBM* + *Axig1pro:HvWUS*, compared to the control with the empty vector (Fig. 7b, d, Table 2). *BBM* has been shown to bind *LAFL* genes (for *LEC1/L1L*, *ABI3*, *FUS3*, and *LEC2*) to regulate their transcription, which places *BBM* upstream of other major regulators for plant embryo identity and totipotency [31]. The effects of *BBM* and *WUS* on regeneration might be explained in part by their regulatory role in genes, such as genes encoding the B3 domain proteins ABSCISIC ACID-INSENSITIVE3 (ABI3) and FUSCA3 (FUS3) (Fig. 7d), suggesting that these TFs might form a feed-forward loop to reinforce cell fate transition. Collectively, we demonstrated that barley *WUS* and *BBM* genes can be used to stimulate barley transformation by enhancing its regenerative potential. On the other hand, overexpression of certain TFs, such as WUS and BBM, has been reported to induce somatic embryogenesis in *Arabidopsis* and maize [31, 42, 45, 54] It will be interesting to investigate the possibility of integrating the effect of BBM and WUS ectopic expression with *Agrobacterium*-mediated barley transformation via direct somatic embryogenesis in our future work.

### Epigenetic reprogramming underlying transcriptome alteration during callus induction

Accumulating evidence has shown that the transcription of many reprogramming genes during callus formation is epigenetically regulated [56, 57]. Genetic mutations or chemical perturbations of epigenetic regulators affect callus formation and shoot regeneration in tissue cultures [58]. The evolutionarily conserved protein complex POLYCOMB REPRESSIVE COMPLEX 2 (PRC2)- mediated histone H3 lysine 27 trimethylation (H3K27me3) is thought to maintain the repressive status of target genes [56], including genes encoding embryonic regulators, such as *LEC2* and *BBM*, to prevent the ectopic onset of embryogenesis and callus formation [59, 60]. A mechanism to activate PRC2-repressed reprogramming regulator genes is to reduce the level of H3K27me3 through histone demethylase. Two candidate genes, HORVU3Hr1G096250 and HORVU7Hr1G073410, encoding proteins similar to *Arabidopsis* SUVH4 and FIS3, respectively, were found to be activated in the two systems (Fig. 8), indicating their regulatory roles in callus induction. Furthermore, HORVU1Hr1G008690, encoding a homolog of SUVH9 in *Arabidopsis*, known as a SET domain protein that acts as a histone methyltransferase, was significantly upregulated in the IME system alone (Fig. 8). We specifically detected the induction of the histone acetylation-related gene *ELP2* (HORVU1Hr1G020620) in the IME system (Fig. 8), raising the possibility that histone acetylation helps activate gene expression. In addition, two histone deacetylase genes, *LSD1* (HORVU6Hr1G078160) and *HDT3B* (HORVU1Hr1G095140), were specifically upregulated in the IME system. This finding is consistent with that of a previous study, which reported that the rice histone deacetylase OsHDA710 regulates callus formation by suppressing repressive OsARFs via histone deacetylation in mature rice embryos [61].

DNA methylation is another important component of epigenetic regulation, and DNA methyltransferase genes display dynamic expression changes after callus induction [32]. Two of them, *DRM2A* (HORVU0Hr1G018360) and *CMT2* (HORVU6Hr1G089250), were significantly activated in the ME system. In contrast, *ELP2* (HORVU1Hr1G020620) was only activated in the IME system (Fig. 8). In addition to DNA methylation and histone modification, auxin has been reported to rewire chromatin accessibility dynamics to promote the acquisition of plant cell totipotency in plant somatic embryogenesis [42]. Further investigation is necessary to understand the molecular link between epigenetic regulation and cell reprogramming during callus formation and shoot regeneration.

## Conclusions

**Fig. 9:**
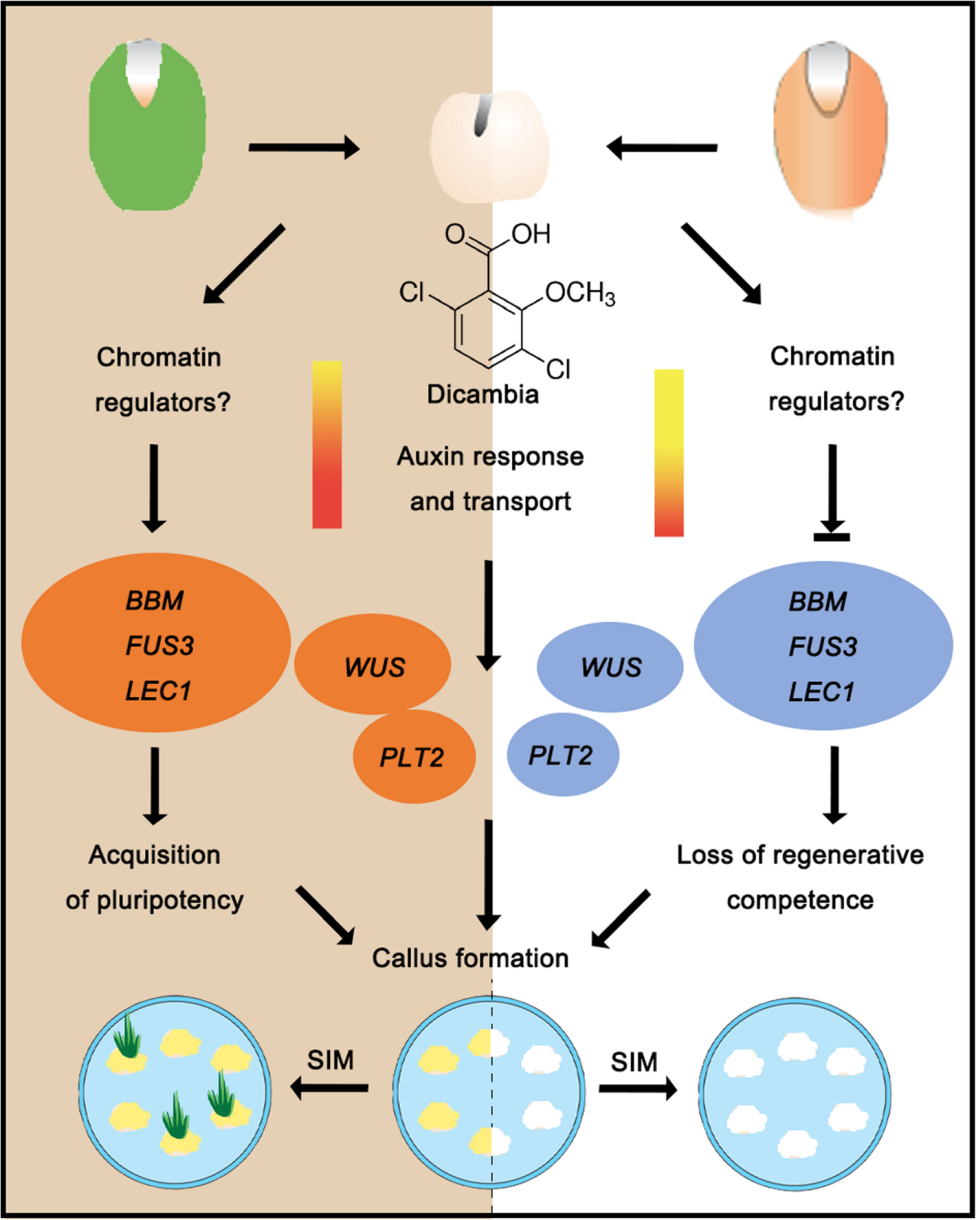
A schematic diagram describing gene expression regulation during callus formation from immature and mature barley embryos. Dicamba (synthetic auxin) induces cell fate transition through the auxin signalling pathway, and more genes are included in the IME system (left) than in the IM system (right). Embryonic genes *BBM*, *LEC1*, and *FUS3*, shoot meristem gene *WUS*, and root meristem gene *PLT2* displayed differential expression patterns between the two systems, resulted in the production of different types of callus. Embryonic callus (left) and non-embryonic callus (right) exhibit differential regeneration potential on shoot-inducing medium (SIM). Orange represents significantly upregulated genes, while blue represents genes that were activated slightly or remained unchanged. Epigenetic modification might be involved in regulating the expression status of regulatory genes in different explants and their responses to callus induction

Through a detailed analysis of gene expression profiles during barley embryo-derived callus induction, we found that more auxin-induced genes were associated with auxin response and transport in the IME system than in the ME system. Embryonic genes *BBM*, *LEC1*, and *FUS3* and the shoot and root meristem genes *WUS* and *PLT2* displayed differential expression patterns between the two systems, indicating their crucial roles in determining the acquisition of competency. Furthermore, epigenetic modifications may participate in regulating the expression of genes in different explants and their responses to callus induction (Fig. 9). *HvBBM* and *HvWUS* might be potential targets for improving barley transformation efficiency.

### Methods

### Plant materials and tissue culture

Barley (*Hordeum vulgare* L. cv. Golden Promise) was grown under natural conditions (from November to May) at the Agricultural Experiment Station of Zhejiang University, Hangzhou, Zhejiang Province, China. The immature seeds (14 days post-pollination) were surface-sterilised for 1 min in 75% (v/v) ethanol, followed by 20 min in 20% (v/v) sodium hypochlorite, and then rinsed five times with sterilised distilled water. The mature seeds were soaked in 50% sulphuric acid for 2 h to remove the seed coat before surface sterilisation. After removing the embryonic axis, IMEs and MEs were placed, with the scutellum facing upward in the callus induction medium [62] in a growth chamber at 24 °C in the dark for callus induction. The MEs and IMEs were cultured in three biological replicates, each replicate consisting of four plates, and each plate containing 30 embryos. The mature embryos were harvested at 0 and 24 h after culture in CIM, while the immature embryos were harvested at 0, 24 h and 48 h. Then they were snap-frozen in liquid nitrogen and stored at − 80 °C until RNA extraction.

### RNA isolation, library construction, and sequencing

A cDNA library was constructed from pooled RNA of immature and mature barley embryos. Using the Illumina HiSeq 4000 paired-end RNA-seq approach, the transcriptome was sequenced, generating a total of 756 million paired-end reads, yielding 114 gigabases (Gb) of sequences. Prior to assembly, the low-quality reads (reads containing sequencing adaptors, reads containing sequencing primers, and nucleotides with a q quality score lower than 20) were removed, resulting in 617 million bp of cleaned, paired-end reads. The raw sequence data have been submitted to the NCBI Short Read database with accession number GSE165487.

The raw sequence data of five samples were aligned to the Ensembl (ftp://ftp.ensemblgenomes.org/pub/release-43/plants/fasta/hordeum_vulgare/dna/Hordeum_vulgare.IBS Cv2.dna.toplevel.fa.gz) barley reference genome using the HISAT package [63], which initially removes a portion of the reads—based on quality information accompanying each read—and then maps the reads to the reference genome. HISAT allows multiple alignments per read (up to 20 by default) and a maximum of two mismatches when mapping the reads to the reference and builds a database of potential splice junctions. This is confirmed by comparing the previously unmapped reads against the database of putative junctions. Then, sequence-dependent bias and amplification noise were removed using UMI-tools [64].

The mapped reads of each sample were assembled using StringTie [65]. Then, all transcriptomes from the samples were merged to reconstruct a comprehensive transcriptome using Perl scripts. After the final transcriptome was generated, StringTie and edgeR were used to estimate the expression levels of all transcripts. StringTie was used to predict mRNA expression levels by calculating FPKM. The differentially expressed mRNAs and genes were selected with |log_2_fold change| ≥1 and with statistical significance of *p* < 0.05, using the R package edgeR [66].

### Real-time qRT-PCR

Total RNA from barley tissues was extracted using RNAiso Plus (TaKaRa, Dalian, China), and 1 μg of RNA was used for first-strand cDNA synthesis using the ReverTra Ace qPCR RT Kit (Toyobo, Shanghai, China). qRT-PCR was performed on the Mastercycler Ep Realplex2 system (Eppendorf, Hamburg, Germany) using a SYBR Green Master Kit (Roche, Basel, Switzerland). The amplification programme was as follows: 10 min 95 °C, and then 10 s at 95 °C, 10 s at 60 °C, 20 s at 72 °C for 40 cycles, followed by a thermal denaturing step. Relative transcript levels were calculated with the ΔΔCt method, using the *ACTIN* gene as a reference. The primer sequences are listed in Additional file 1: Table S4.

### Vector construction and *Agrobacterium*-mediated barley transformation

The arrangement of expression cassettes within the T-DNA of plasmids used in this study is shown in Fig. 7a. The *proZmPLTP:ZmBBM + proZmAxig1:ZmWUS2* construct contained two cassettes: the first one included a maize phospholipid transferase promoter (*proZmPLTP*) driving *ZmBBM* with a Nos terminator, and the second one included a maize *Axig1* promoter (*proZmAxig1*) driving *WUS2* with a Nos terminator [30]. For the *proZmPLTP:HvBBM + proZmAxig1:HvWUS2* construct, the promoters used were identical to those in the *proZmPLTP:ZmBBM + proZmAxig1:ZmWUS2* construct, with the homologous genes in barley replacing *ZmBBM* and *ZmWUS2*. All the promoters and genes were amplified by PCR, and the PCR products were assembled using an infusion kit (TaKaRa, Dalian, China), and then sub-cloned into *pCAMBIA1305*. The primers used in this study are listed in Additional file 1: Table S3. The clones used for vector construction were verified using sequencing. The constructs described were electroporated into *Agrobacterium tumefaciens* strain EHA105. Caryopses were harvested 2–3 weeks after pollination. Immature scutella, 1.5–2 mm in size, were obtained from barley embryos after removal of the embryo axis and used as explants for *Agrobacterium*-mediated transformation following the procedure of Harwood (2014) [62]. Transgenic calli were induced from infected immature scutella on hygromycin (50 mg L^− 1^) containing medium, and plantlets resistant to hygromycin were regenerated. Regenerated plants at the seedling stage were grown for 12–16 weeks in a growth chamber with a 16 h light/8 h night cycle, a temperature of 23 °C and 70% humidity. Subsequently, transgenic plants were grown until maturity under natural light in 6-in. pots in a glasshouse [62]. Transformation frequency was defined as the number of treated immature embryos that produced hygromycin-resistant T_0_ plants.

## Supplementary Information

**Additional file 1: Table S1.** Statistics of the total reads from five libraries. Sample: sample name; Raw_reads: total number of reads in raw data; Raw_bases (G): total data volume of offline raw data; Valid_reads: number of valid reads after removing joints, low-quality reads, etc.; Dedup_reads: the number of reads after UMI deduplication; Valid_Q20 (%): Q20 value of valid reads; Valid_Q30 (%): Q30 value of valid reads; Valid_GC (%): GC content of reads after UMI deduplication; Valid2raw (%): valid reads accounted for the percentage of raw reads; Dedup2Valid (%): the percentage of valid reads after deduplication of the reads in the genome. **Table S2.** Summary of data cleaning and length distribution of tags. **Table S3.** List of primers for plasmid construction. The parts marked in red represents the adapter sequences. Fw: forward primer. Rev.: reverse primer. **Table S4.** List of primers used in the qRT-PCR. F: forward primer. R: reverse primer. **Fig. S1.** Pearson correlation between samples. **a:** Correlation heat map between samples. **b:** Principal component analysis of the three-dimensional map. **Fig. S2.** Fig. S2 Expression of a set of callus-inducing medium (CIM)--induced transcription factors during immature and mature embryo-derived callus formation. **a-b:** Pie chart of differentially expressed transcription factors in IME_0h/IME_48h and ME_0h/ME_24h. Numbers represent the gene members associated with a given TF family. **c:** The top 10 differentially expressed TFs in IME_0h/IME_48h ranked by fold change. Genes marked in blue are upregulated TFs, and transcription factors marked in black are downregulated. **d:** The top 10 differentially expressed TFs in ME_0h/ME_24h. **Fig. S3.** Transcript levels of *ARF11*, *ARF16B*, *SUVH2A* and *SUVH3A* in the five samples as revealed by qRT-PCR and RNA-seq data. The data shown are means ± S.D. of three biological replicates. **Fig. S4.** Phylogenetic tree of *BBM* and *WUS* genes in barley and other species. The phylogenetic tree was constructed in MEGA 4 by the Neighbor-Joining method. The gene IDs are *HvBBM* (HORVU2Hr1G087310.1), *HvWUS* (HORVU3Hr1G085050.1) from *Hordeum vulgare*, *ZmBBM* (Zm00001d042492), *ZmWUS* (Zm00001d026537) from *Zea mays*, *SbBBM* (SORBI_3003G390600), *SbWUS* (SORBI_3006G254900) from *Sorghum bicolor*, *OsBBM* (LOC_Os01g67410.1), *OsWUS* (LOC_Os04g56780.1) from *Oryza sativa*, *TaBBM* (TraesCS3B02G427300.1), *TaWUS* (TraesCS2A02G491900.1) from *Triticum aestivum*, *BdBBM* (BRADI_2g57747v3), *BdWUS* (BRADI_5g25113v3) from *Brachypodium distachyon*, and *AtBBM* (AT5G17430.1), *AtWUS* (AT2G17950.1) from *Arabidopsis thaliana*. **Fig. S5.** Expression analysis of candidate *LEC1* gene during callus formation. **a:** Sequence alignment and domain analysis of the LEC1 in *Arabidopsis*, rice, maize and barley. **b:** Phylogenetic tree of LEC1 among barley and other species. The phylogenetic tree was constructed in MEGA 4 by the Neighbor-Joining method. The gene IDs are *HvLEC1* (HORVU6Hr1G072110) from *Hordeum vulgare*, *AtLEC1* (AT1G21970.1) from *Arabidopsis thaliana*, *BnLEC1* (BnaA07g10770D) from *Brassica napus*, *GmLEC1-A* (GLYMA_07G268100), *GmLEC1-B* (GLYMA_17G005600) from *Glycine max,* and *ZmLEC1* (Zm00001d017898_T001) from *Zea mays*, and *OsLEC1* (LOC_Os02g49370.1) from *Oryza sativa*. **Fig. S6.** Phenotypes of plants regenerated from callus transformed with empty vectors and with *WUS* and *BBM* genes. **Fig. S7.** Expression analysis of candidate *LBD* genes potentially associated with callus formation in barley. The expression levels were visualised by using OmicStudio tools at https://www.omicstudio.cn/tool based on RNA-seq datasets (Additional file 4). Numbers beneath the heat map indicate the relative expression intensities, and the higher expression intensities are indicated by more reddish colors.

**Additional file 2.** Supplementary raw data of DEGs.

**Additional file 3.** Supplementary raw data of TFs.

**Additional file 4.** Supplementary raw data for genes in heatmap.

## Data Availability

All data generated or analysed during this study are included in this published article and its supplementary information files.

## Abbreviations

IME: immature embryo
ME: mature embryo
DEGs: differentially expressed genes
CIM: callus inducing medium
SIM: shoot inducing medium
WOX5: WUSCHEL-RELATED HOMEOBOX5
SHR: SHORT ROOT
ARF: AUXIN RESPONSE FACTOR
LBD: LATERAL ORGAN BOUNDARIES DOMAIN
CUC: CUP-SHAPED COTYLEDON
PLTs: PLETHORA proteins
ABI3: ABSCISIC ACID-INSENSITIVE 3
FUS3: FUSCA3
PRC2: POLYCOMB REPRESSIVE COMPLEX 2
SAM: shoot apical meristem
RAM: root apical meristem
DPA: days post-anthesis
qRT-PCR: quantitative reverse transcription PCR
GO: gene ontology
UMI: unique molecular identifier
TFs: transcription factors
BBM: BABY BOOM
WUS: WUSCHEL
LEC1: LEAFY COTYLEDON 1
IAA: INDOLEACETIC ACID
SAUR: SMALL AUXIN UP RNA
PIN: PIN-FORMED
YUC: YUCCA
