# Supplementary material for: Identification of regulatory factors promoting embryogenic callus formation in barley through transcriptome analysis
Source: BMC Plant Biol. 2021 Mar 19;21:145. doi: 10.1186/s12870-021-02922-w (PMC7980361; doi:10.1186/s12870-021-02922-w)
Supplement: Supplementary file 1 — Additional file 1: Table S1. Statistics of the total reads from five libraries. Sample: sample name; Raw_reads: total number of reads in raw data; Raw_bases (G): total data volume of offline raw data; Valid_reads: number of valid reads after removing joints, low-quality reads, etc.; Dedup_reads: the number of reads after UMI deduplication; Valid_Q20 (%): Q20 value of valid reads; Valid_Q30 (%): Q30 value of valid reads; Valid_GC (%): GC content of reads after UMI deduplication; Valid2raw (%): valid reads accounted for the percentage of raw reads; Dedup2Valid (%): the percentage of valid reads after deduplication of the reads in the genome. Table S2. Summary of data cleaning and length distribution of tags. Table S3. List of primers for plasmid construction. The parts marked in red represents the adapter sequences. Fw: forward primer. Rev.: reverse primer. Table S4. List of primers used in the qRT-PCR. F: forward primer. R: reverse primer. Fig. S1. Pearson correlation between samples. a: Correlation heat map between samples. b: Principal component analysis of the three-dimensional map. Fig. S2. Fig. S2 Expression of a set of callus-inducing medium (CIM)--induced transcription factors during immature and mature embryo-derived callus formation. a-b: Pie chart of differentially expressed transcription factors in IME_0h/IME_48h and ME_0h/ME_24h. Numbers represent the gene members associated with a given TF family. c: The top 10 differentially expressed TFs in IME_0h/IME_48h ranked by fold change. Genes marked in blue are upregulated TFs, and transcription factors marked in black are downregulated. d: The top 10 differentially expressed TFs in ME_0h/ME_24h. Fig. S3. Transcript levels of ARF11, ARF16B, SUVH2A and SUVH3A in the five samples as revealed by qRT-PCR and RNA-seq data. The data shown are means ± S.D. of three biological replicates. Fig. S4. Phylogenetic tree of BBM and WUS genes in barley and other species. The phylogenetic tree was constructed in MEGA 4 by the [file 12870_2021_2922_MOESM1_ESM.pdf]

Full title:

Identification of regulatory factors promoting embryogenic callus formation in barley through transcriptome analysis

Authors:

Jingqi Suo, Chenlu Zhou, Zhanghui Zeng, Xipu Li, Hongwu Bian, Junhui Wang, Muyuan Zhu and Ning Han*

Additional file 1

Table S1. Statistics of the total reads from 5 libraries.

Sample		Raw_ reads	Raw_ Bases (G)	Valid_ reads	Dedup _reads	Valid_ Q20 (%)	Valid_ Q30 (%)	Valid_ GC (%)	Valid 2raw (%)	Dedup 2Valid (%)
IME_0h	1	51193136	7.68	50508092	41238767	99.94%	97.65%	54.0%	98.66%	81.65%
	2	51358534	7.70	50725082	41505310	99.94%	97.71%	54.0%	98.77%	81.82%
	3	52190674	7.83	51346982	41532379	99.94%	97.68%	54.0%	98.38%	80.89%
IME_24h	1	43390274	6.51	42482012	36191633	99.93%	97.49%	51.0%	97.91%	85.19%
	2	47504050	7.13	46361390	38961715	99.94%	97.51%	51.5%	97.59%	84.04%
	3	50787806	7.62	49289192	41412312	99.94%	97.43%	51.5%	97.05%	84.02%
IME_48h	1	51336244	7.70	50064794	41202782	99.92%	97.54%	51.5%	97.52%	82.30%
	2	54437730	8.17	52617624	44415455	99.94%	97.59%	51.0%	96.66%	84.41%
	3	53048612	7.96	51022778	43251413	99.94%	97.47%	51.5%	96.18%	84.77%
ME_0h	1	45827838	6.87	44726072	37049023	99.94%	97.43%	51.5%	97.60%	82.84%
	2	50216544	7.53	48998490	40579584	99.94%	97.40%	51.5%	97.57%	82.82%
	3	53234728	7.99	51294828	42127157	99.93%	97.65%	51.5%	96.36%	82.13%
ME_24h	1	51008886	7.65	49828700	42149102	99.93%	97.62%	51.5%	97.69%	84.59%
	2	53514406	8.03	51117256	43701976	99.94%	97.64%	51.5%	95.52%	85.49%
	3	50870118	7.63	49956734	42714327	99.93%	97.60%	51.5%	98.20%	85.50%

Sample: sample name; Raw_reads: total number of reads in raw data; Raw_bases (G): total data volume of offline raw data; Valid_reads: number of valid reads after removing joints, low-quality reads, etc.; Dedup_reads: the number of reads after UMI deduplication; Valid_Q20 (%): Q20 value of valid reads; Valid_Q30 (%): Q30 value of valid reads; Valid_GC (%): GC content of reads after UMI deduplication; Valid2raw (%): valid reads accounted for the percentage of raw reads; Dedup2Valid (%): the percentage of valid reads after deduplication of the reads in the genome.

Table S2. Summary of data cleaning and length distribution of tags

**length range**	**transcripts**	**genes**
>=20000	3 (0.00%)	396 (2.73%)
10000-20000	251 (0.15%)	611 (4.21%)
5000-10000	6,100 (3.66%)	1,803 (12.41%)
2000-5000	61,432 (36.82%)	4,537 (31.23%)
1000-2000	63,663 (38.16%)	3,484 (23.98%)
500-1000	25,153 (15.08%)	1,874 (12.90%)
300-500	6,025 (3.61%)	778 (5.35%)
<300	4,222 (2.53%)	1,046 (7.20%)
total number	166,849	14,529

Table S3 List of primers for plasmid construction.

Name	Sequence (5'-3')
Fw-Axig1	TATGACCATGATTACGAATTTTCTTCATCATCCTCCCCAG
Rev-Axig1	GTGACCTCTCTCACTTTCCCTTGATCAGCC
Fw-HvWUS	GGGAAAGTGAGAGAGGTCACTAGCACAACAAAGTGGGGGT
Rev-HvWUS	ATGGCCGCGGGGACAATGACATCAACGAAAACGGGGTGGA
Fw-Tnos	TTGATGTCATTGTCCCCGCGGCCATGCTAGAGTCC
Rev-Tnos	ATGTCGATAGGTCACTGGATTTTGGTTT
Fw-PLTP	CCAGTGACCTATCGACATGTGGGCTCCATT
Rev-PLTP	GAATGATTCGGTCGCCACTGCCAACTTCT
Fw-HvBBM	CAGTGGCGACCGAATCATTCGCTAGCTTTGACTGCCCTGA
Rev-HvBBM	CCAGTGACCTTTAAGTGTCGAGTCCTCCTACTTCACCCGT
Fw-PolyA	CGACACTTAAAGGTCACTGGATTTTGGTTT
Rev-PolyA	CTTGCATGCCTGCAGGTCGACCGCGGCCATGCTAGAGTCC
Fw-ZmWUS	AGTGAATGGCGGCCAGAGAGGTCACTGCACAGGAG
Rev- ZmWUS	CGCGGTCACATGCTCGGACAATGACAGCAACGCAC
Fw-ZmBBM	CTCCTCAAGGCGAATCAATCTAAGAAGAAACTCAA
Rev-ZmBBM	CCCGGGGATCTTAAGTGTCGTTCCAGACAC

The parts marked in red represents the adapter sequences. Fw: forward primer. Rev: reverse primer.

Table S4 List of primers used in the qRT-PCR.

Name	Sequence (5'-3')
QPCR-ACTIN-F	GCTGAGCGGGAAATTGTAAG
QPCR-ACTIN-R	GATCATGGATGGCTGGAAGA
QPCR-LEC1-F	TACGCGCCAGGAAATAGTGG
QPCR-LEC1-R	CCGAAGGTCTGGTGGTTCTC
QPCR-PLT5-F	GGGGTTCGGGCCAGTAATTT
QPCR-PLT5-R	CACCCCGCGAAGATGTAGAA
QPCR-BBM-F	CAGTGGTTTTTCTCGTGGCG
QPCR-BBM-R	GCGACTCTCCCTATCCTTGC
QPCR-WUS-F	CCATCCCCGGATCATGACATA
QPCR-WUS-R	CAGGTCGTAGGCTGCTTTGA
QPCR-ARF11-F	TCGTCACGTCAAAGAAGCTGA
QPCR-ARF11-R	CCTGGGCCTGTAGTAGACCA
QPCR-ARF16B-F	TGGTGTACAATGCCAGACACA
QPCR-ARF16B-R	TGGAAAACCAAGCAATCGCC
QPCR-PIN1A-F	GGATCTTCACGCCGGACCAG
QPCR-PIN1A-F	GAGATGAAGTGGAAGGACAGCA
QPCR-SUVH2A-F	ACCTTGCTCTCGAGAGGAGT
QPCR-SUVH2A-R	GCTGTGTCCTCTTCCATATC
QPCR-SUVH3A-F	ACCTTGCTCTCGAGAGGAGT
QPCR-SUVH3A-R	GCTGTGTCCTCTTCCATATT

F: forward primer. R: reverse primer.

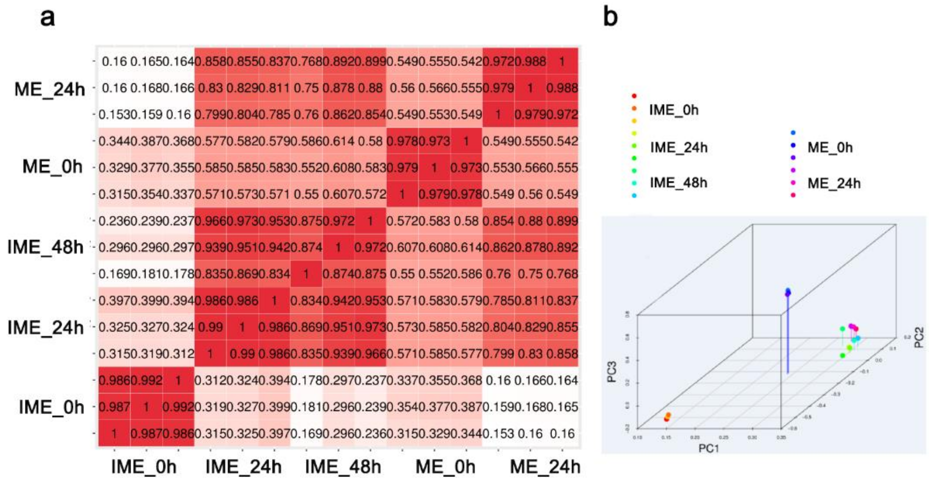

Fig. S1 Pearson correlation between samples. **a:** Correlation heat map between samples. **b:** Principal component analysis of the three-dimensional map.

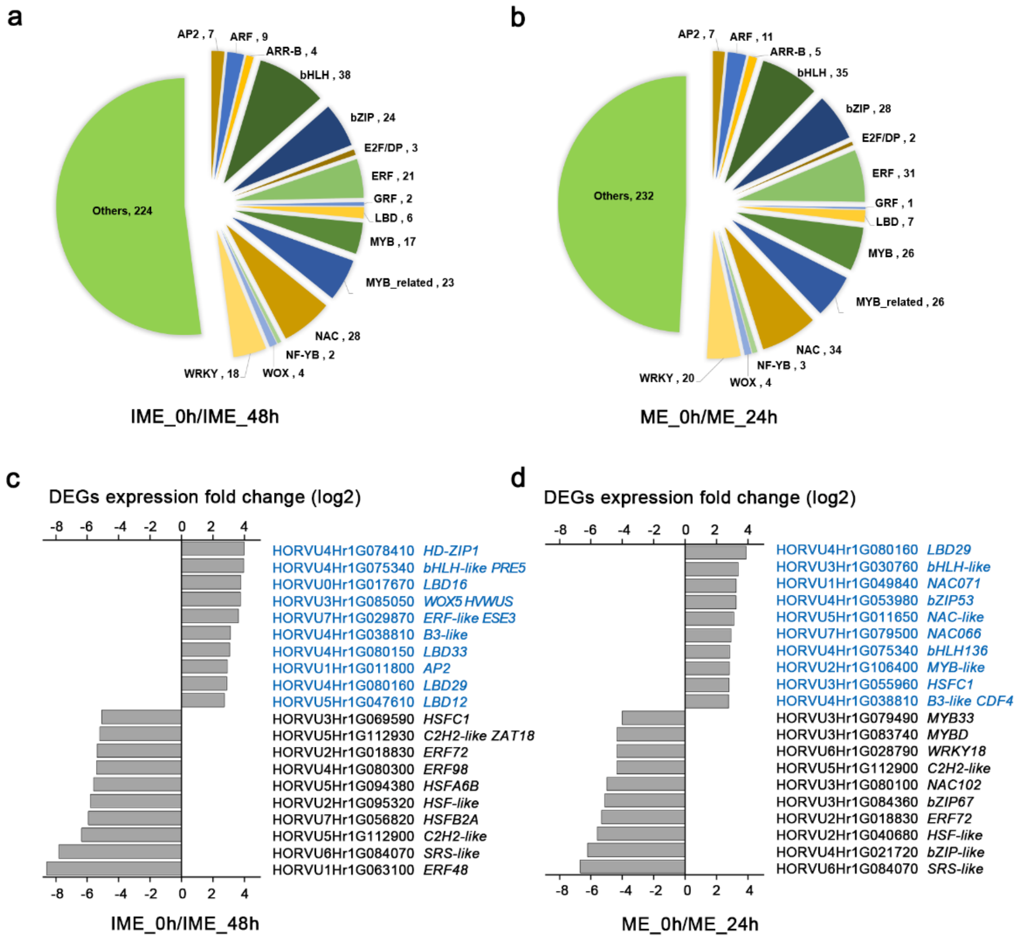

Fig. S2 Expression of a set of callus-inducing medium (CIM)--induced transcription factors during immature and mature embryo-derived callus formation. **a-b:** Pie chart of differentially expressed transcription factors in IME_0h/IME_48h and ME_0h/ME_24h. Numbers represent the gene members associated with a given TF family. **c:** The top 10 differentially expressed TFs in IME_0h/IME_48h ranked by fold change. Genes marked in blue are upregulated TFs, and transcription factors marked in black are downregulated. **d:** The top 10 differentially expressed TFs in ME_0h/ ME_24h.

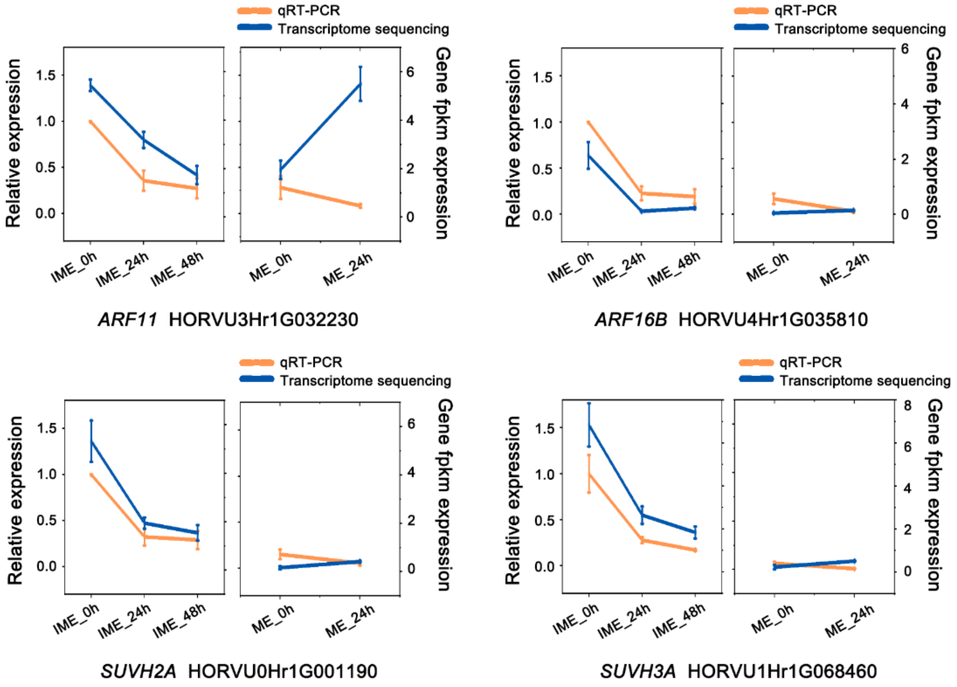

Fig. S3 Transcript levels of *ARF11*, *ARF16B*, *SUVH2A* and *SUVH3A* in the five samples as revealed by qRT-PCR and RNA-seq data. The data shown are means ± S.D. of three biological replicates.

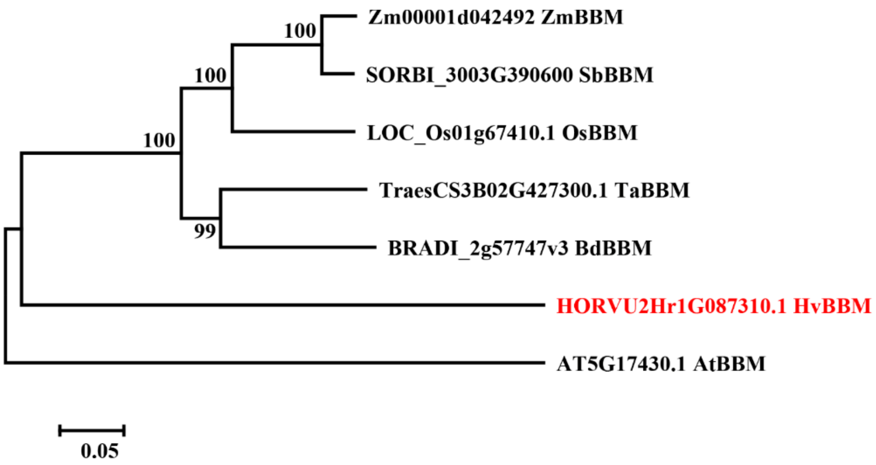

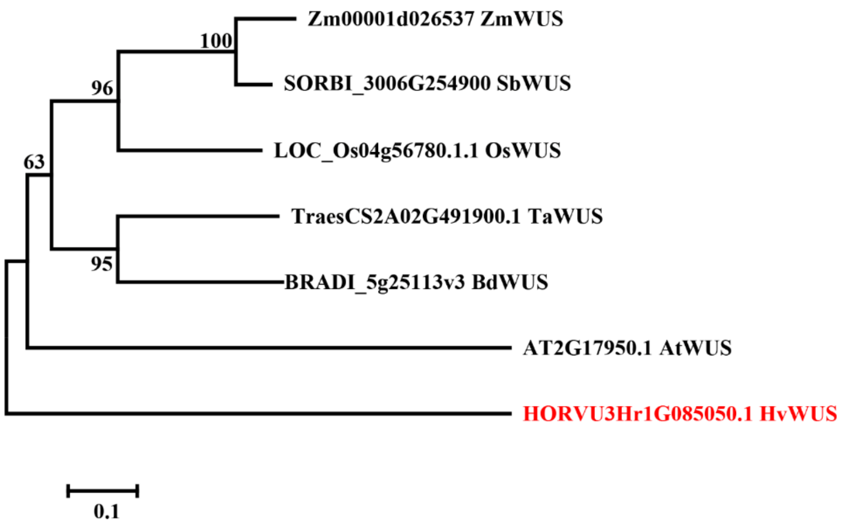

Fig. S4 Phylogenetic tree of *BBM* and *WUS* genes in barley and other species. The phylogenetic tree was constructed in MEGA 4 by the Neighbor-Joining method. The gene IDs are *HvBBM* (HORVU2Hr1G087310.1), *HvWUS* (HORVU3Hr1G085050.1) from *Hordeum vulgare*, *ZmBBM* (Zm00001d042492), *ZmWUS* (Zm00001d026537) from *Zea mays*, *SbBBM* (SORBI_3003G390600), *SbWUS* (SORBI_3006G254900) from *Sorghum bicolor*, *OsBBM* (LOC_Os01g67410.1), *OsWUS* (LOC_Os04g56780.1) from *Oryza sativa*, *TaBBM* (TraesCS3B02G427300.1), *TaWUS* (TraesCS2A02G491900.1) from *Triticum aestivum*, *BdBBM* (BRADI_2g57747v3), *BdWUS* (BRADI_5g25113v3) from *Brachypodium distachyon*, and *AtBBM* (AT5G17430.1), *AtWUS* (AT2G17950.1) from *Arabidopsis thaliana*.

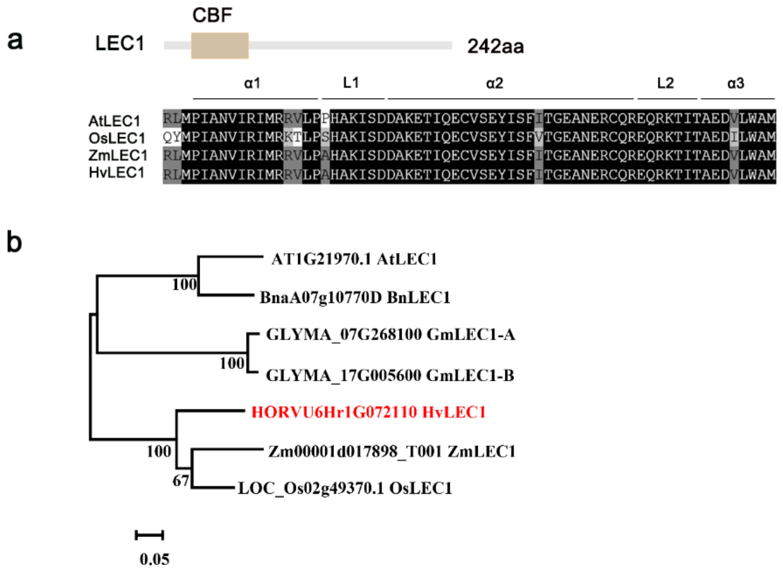

Fig. S5 Expression analysis of candidate *LEC1* gene during callus formation. **a:** Sequence alignment and domain analysis of the LEC1 in *Arabidopsis*, rice, maize and barley. **b:** Phylogenetic tree of LEC1 among barley and other species. The phylogenetic tree was constructed in MEGA 4 by the Neighbor-Joining method. The gene IDs are *HvLEC1* (HORVU6Hr1G072110) from *Hordeum vulgare*, *AtLEC1* (AT1G21970.1) from *Arabidopsis thaliana*, *BnLEC1* (BnaA07g10770D) from *Brassica napus*, *GmLEC1-A* (GLYMA_07G268100), *GmLEC1-B* (GLYMA_17G005600) from *Glycine max*, and *ZmLEC1* (Zm00001d017898_T001) from *Zea mays*, and *OsLEC1* (LOC_Os02g49370.1) from *Oryza sativa*.

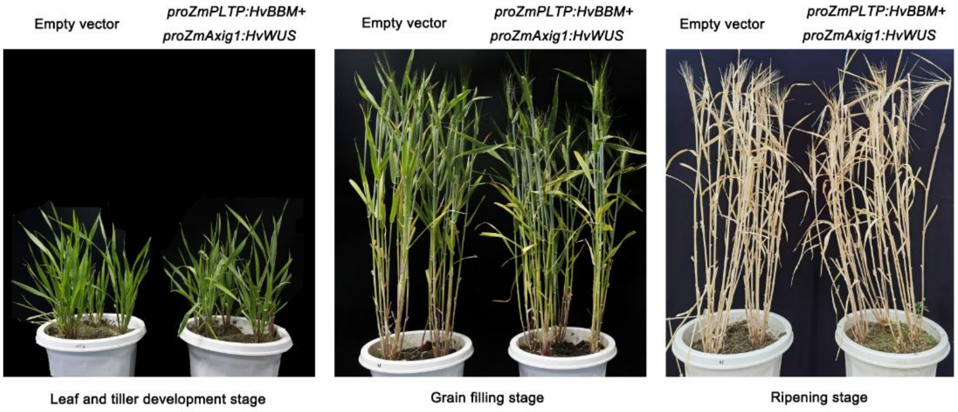

Fig. S6 Phenotypes of plants regenerated from callus transformed with empty vectors and with *WUS* and *BBM* genes.

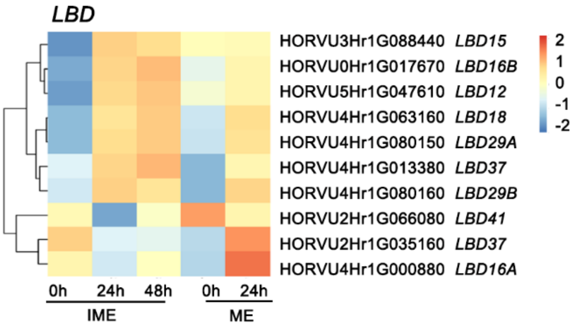

Fig. S7 Expression analysis of candidate *LBD* genes potentially associated with callus formation in barley. The expression levels were visualised by using OmicStudio tools at https://www.omicstudio.cn/tool based on RNA-seq datasets (Additional file 4). Numbers beneath the heat map indicate the relative expression intensities, and the higher expression intensities are indicated by more reddish colors.
